# Supplementary figures and images for: Genetic dissection of a Leishmania flagellar proteome demonstrates requirement for directional motility in sand fly infections
Source: PLoS Pathog. 2019 Jun 26;15(6):e1007828. doi: 10.1371/journal.ppat.1007828 (PMC6615630; doi:10.1371/journal.ppat.1007828)

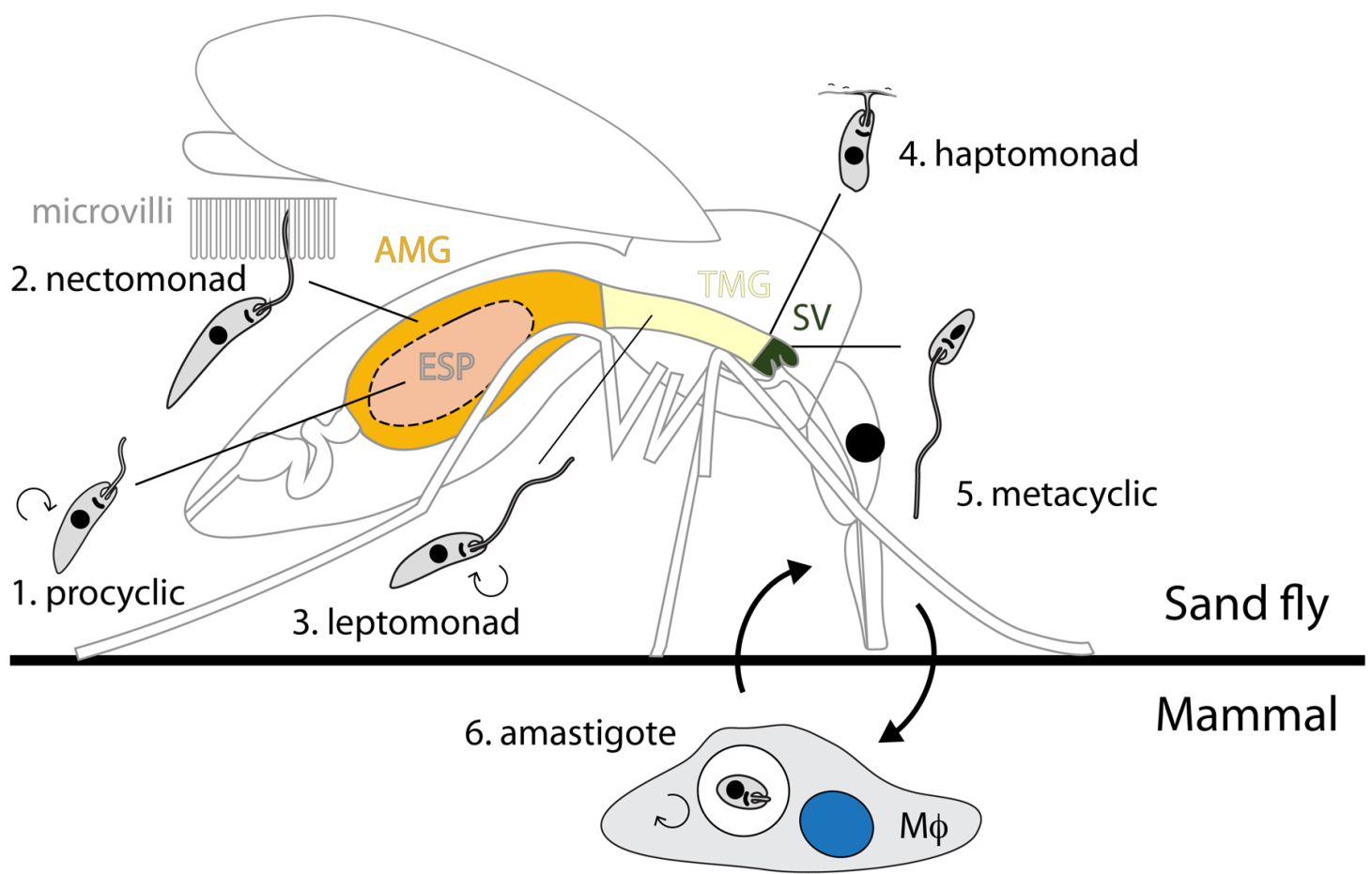

S1 Figure

Supplement: S1 Fig — Overview of the different developmental forms described for L. mexicana promastigotes (1–5) and their locations in the sand fly (drawn after descriptions in [22,99]), and amastigotes (6) in mammalian macrophages. Replicative forms are indicated with a curved arrow. Nectomonad promastigotes adhere via their flagella to the gut microvilli, haptomonads are attached to the chitin lining of the stomodeal valve. ESP: endoperitrophic space, AMG: abdominal midgut, TMG: thoracic midgut, SV: stomodeal valve, MΦ: macrophage. (PDF) [file ppat.1007828.s011.pdf]

**A**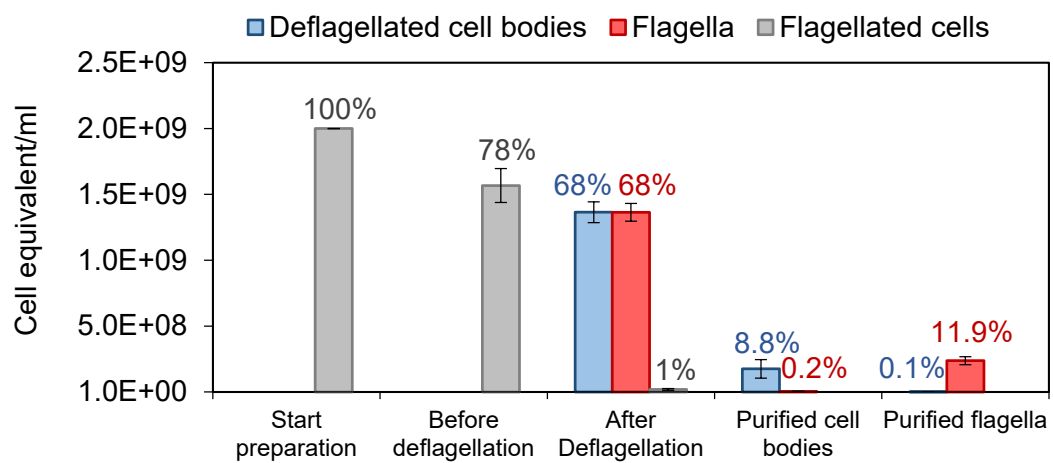**B**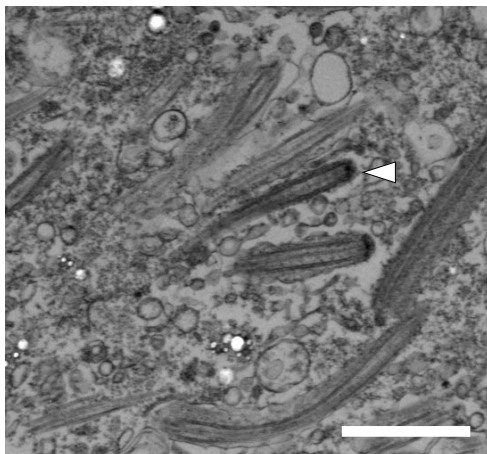**C**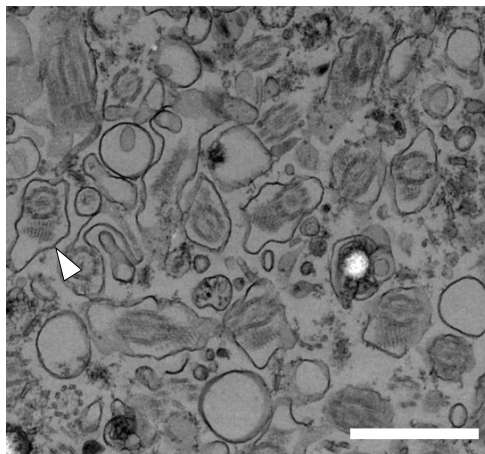**D**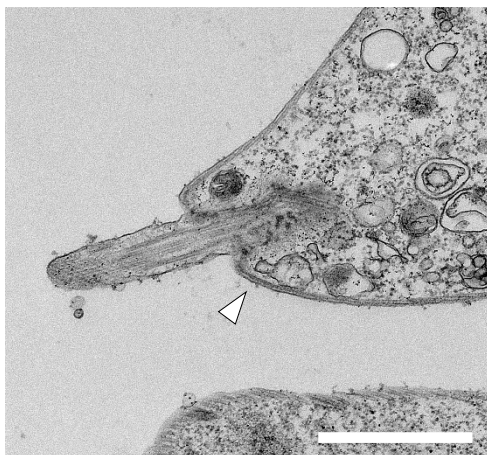**E**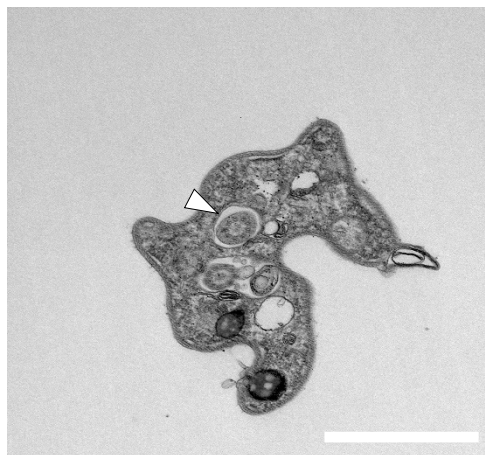

S2 Figure

Supplement: S2 Fig — (A) Quantitation of yield and purity. Counts of whole flagellated cells, isolated flagella or deflagellated cell bodies for each stage of the deflagellation procedure. The purified cell body fraction contains 2.3% isolated flagella; the purified flagella fraction contains 0.84% deflagellated cell bodies. Error bars represent standard deviations between four biological replicates. (B,C) Transmission electron microscopy of isolated flagella. Arrows indicate flagellar tip structure (B); cross-section through flagellum with intact axoneme, associated PFR and surrounding membrane (C). (D,E) Transmission electron microscopy of deflagellated cell bodies. Arrows point to the anterior end of a deflagellated cell body (D) and intact axonemal structure of flagellum inside the flagellar pocket (E). Scale bars represent 1 μm. (PDF) [file ppat.1007828.s012.pdf]

A

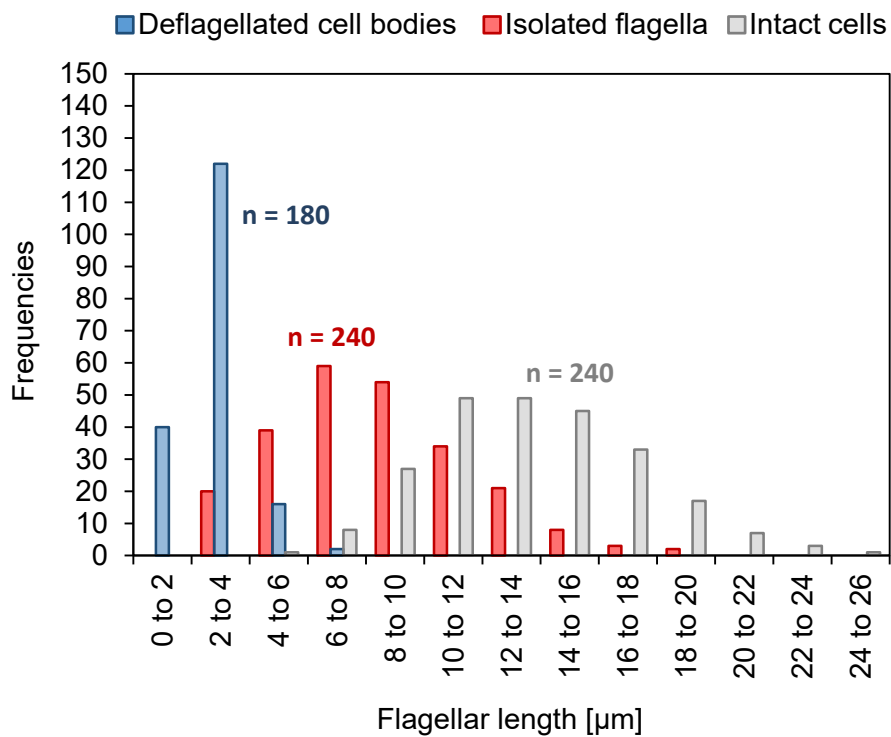

B

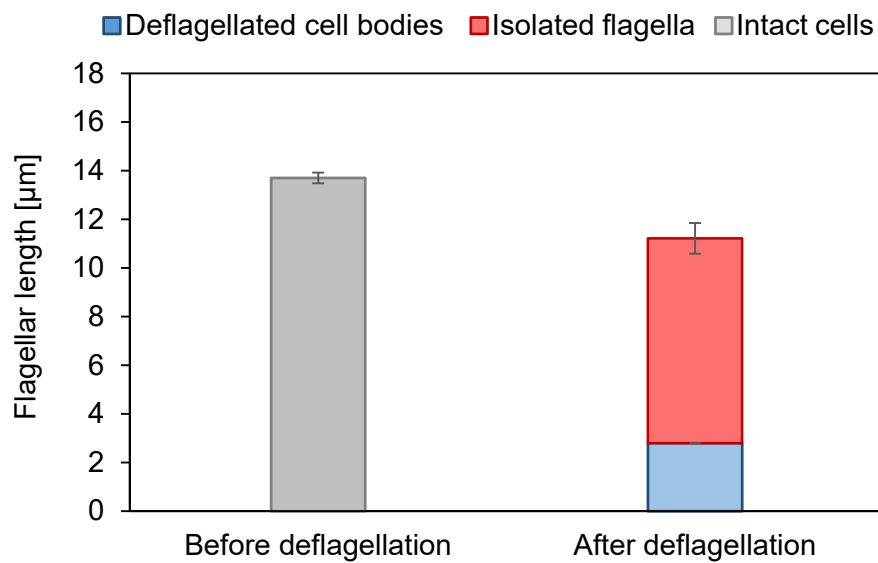

C

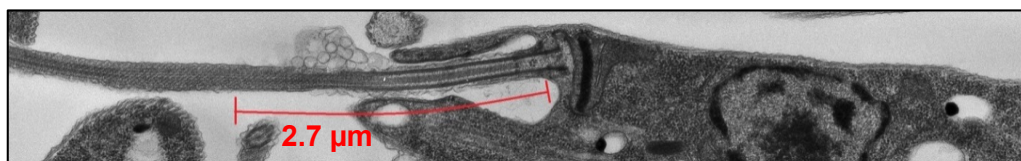

S3 Figure

Supplement: S3 Fig — (A) Measurements of flagellar lengths for intact cells, isolated flagella or deflagellated cell bodies from four independent samples. (B) Average lengths derived from data shown in (A). The length measurements of the cell body fractions and flagella fractions are stacked to show the combined total length after deflagellation. Combined standard deviation for isolated flagella and deflagellated cell bodies is calculated by z=x2+y2. (C) Electron micrograph of an intact L. mexicana promastigote cell. The red line shows the average length of flagellum remaining attached to the cell body. (PDF) [file ppat.1007828.s013.pdf]

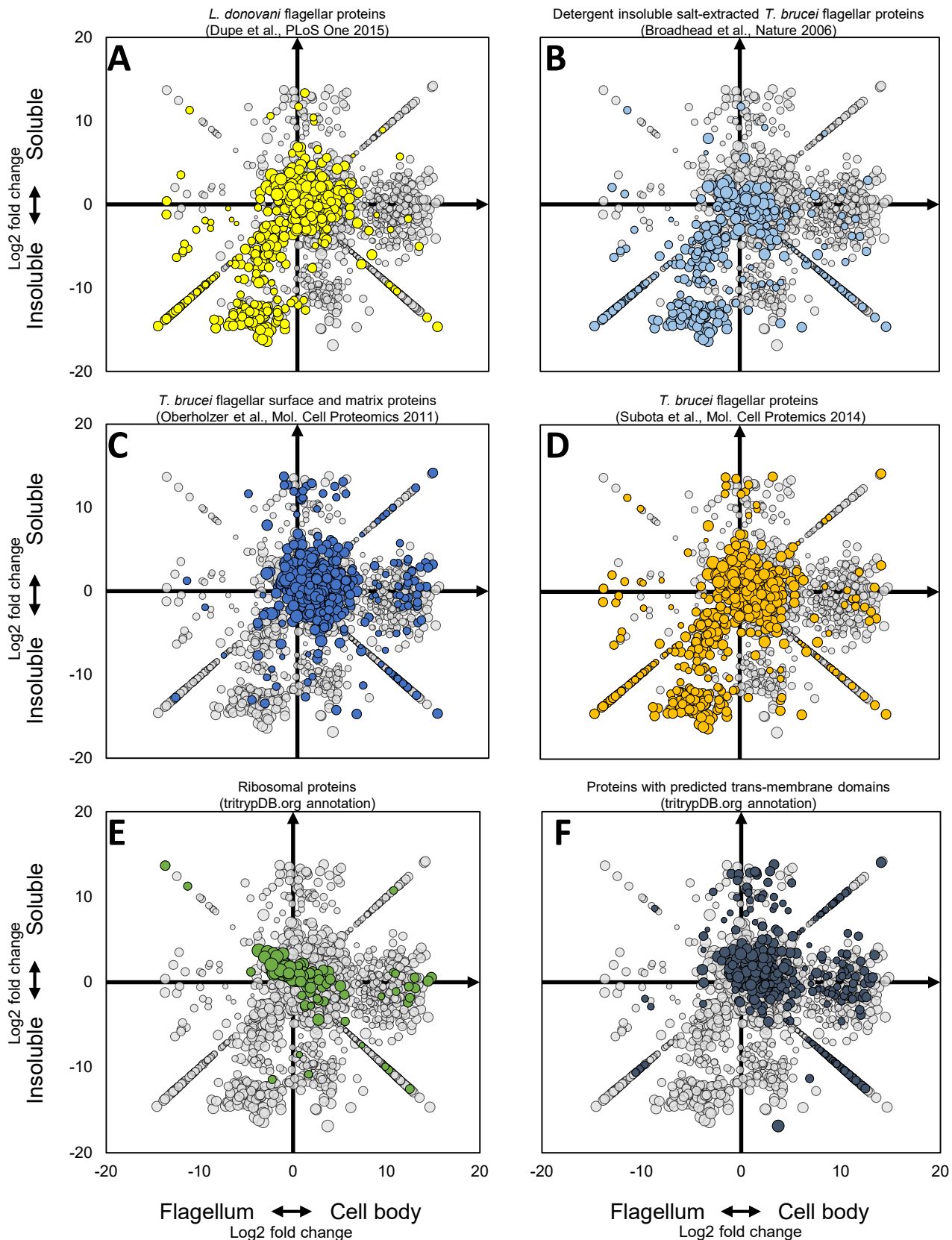

S5 Figure

Supplement: S5 Fig — All 2414 proteins detected in run 1 were plotted as in Fig 2A. Highlighted in colour are (A) orthologs of L. donovani flagellar proteins [100], (B) orthologs of detergent insoluble salt-extracted T. brucei flagellar proteins [6], (C) orthologs of T. brucei flagellar surface and matrix proteins [85], (D) orthologs of T. brucei proteins detected in mechanically sheared flagella [101], (E) L. mexicana ribosomal proteins and (F) L. mexicana proteins with trans-membrane domain predictions. Each plot can be interactively explored on http://www.leishgedit.net/leishgedit_db/. (PDF) [file ppat.1007828.s015.pdf]

A

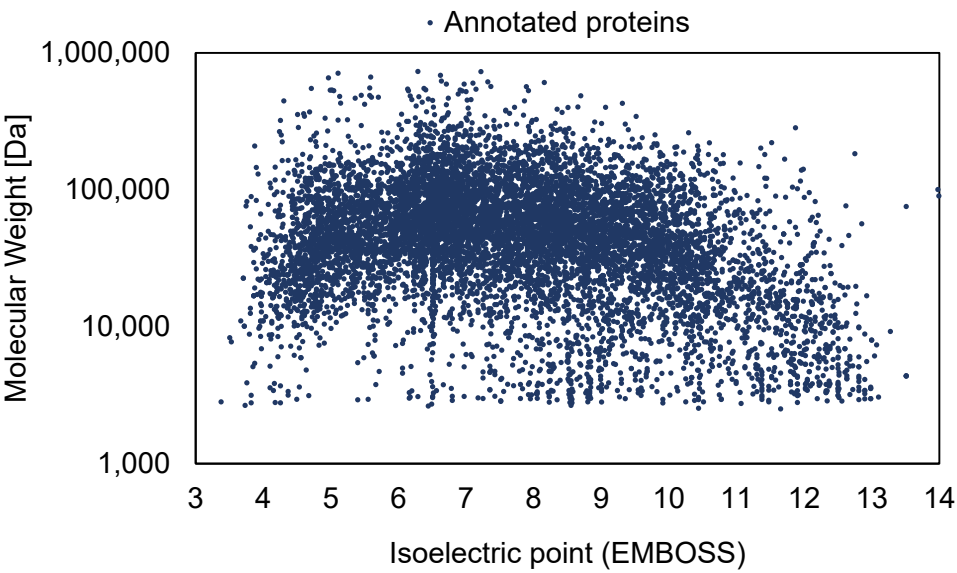

B

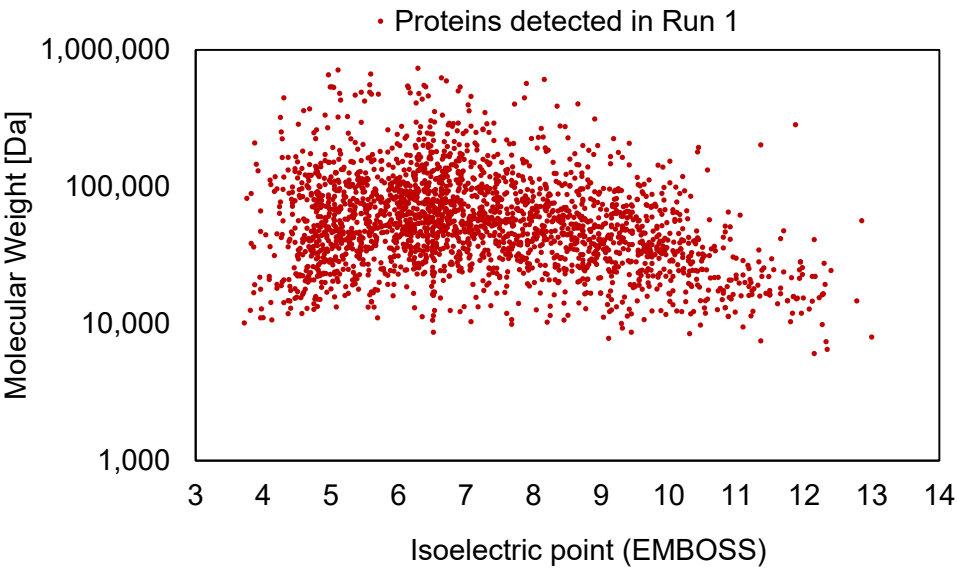

S6 Figure

Supplement: S6 Fig — Molecular weight and isoelectric point calculated with Isoelectric Point Calculator [102]. The isoelectric point prediction model from EMBOSS is shown. (A) All annotated proteins, based on gene models from [29]. (B) Proteins detected in MS run 1. Two sample Kolmogorov–Smirnov test shows significant difference in distributions between (A) and (B) (p-value = 9.63e-30). (PDF) [file ppat.1007828.s016.pdf]

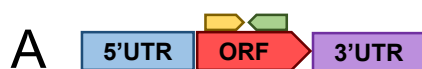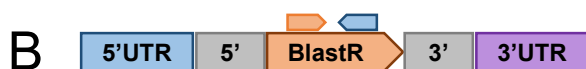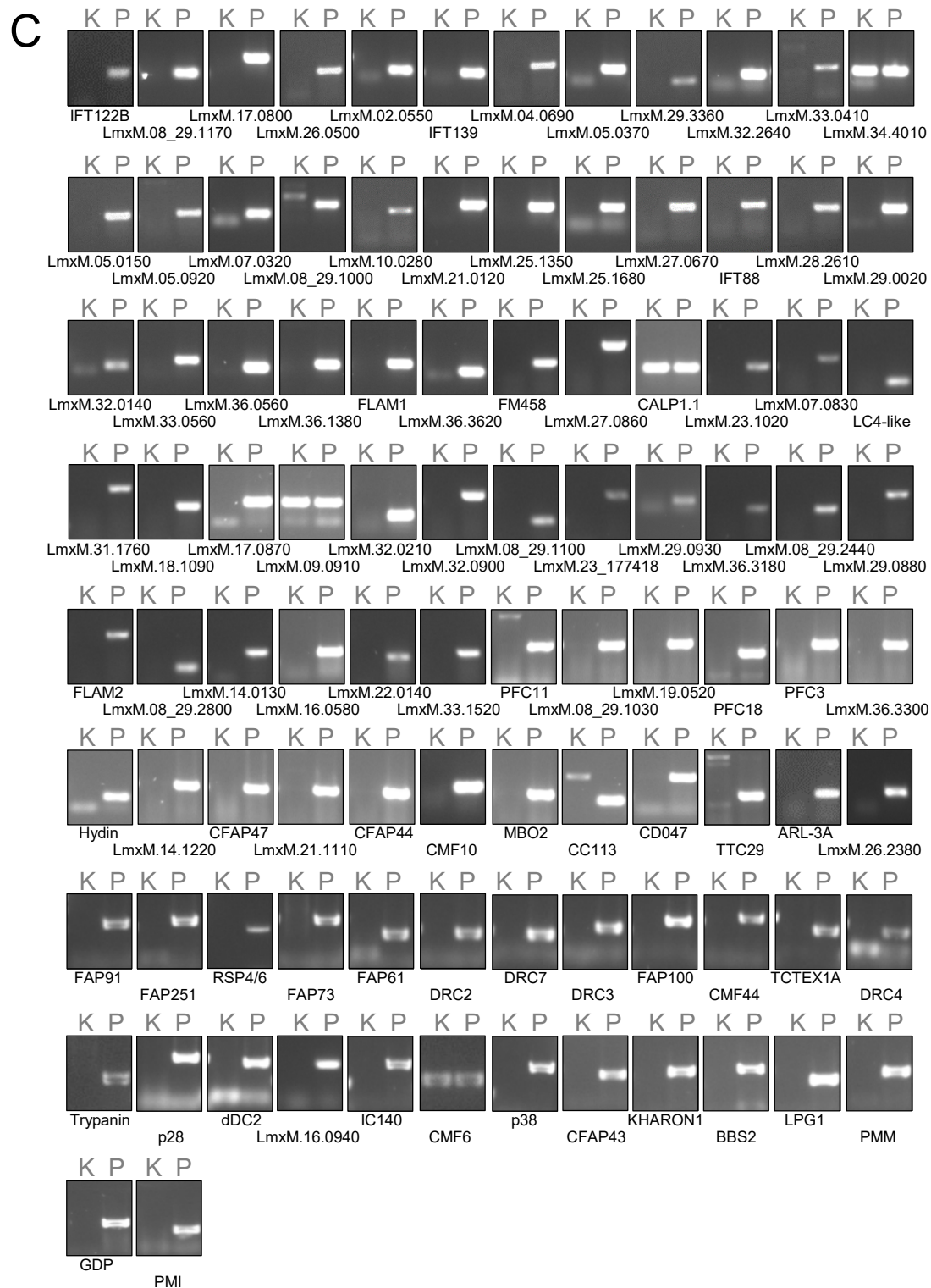

D

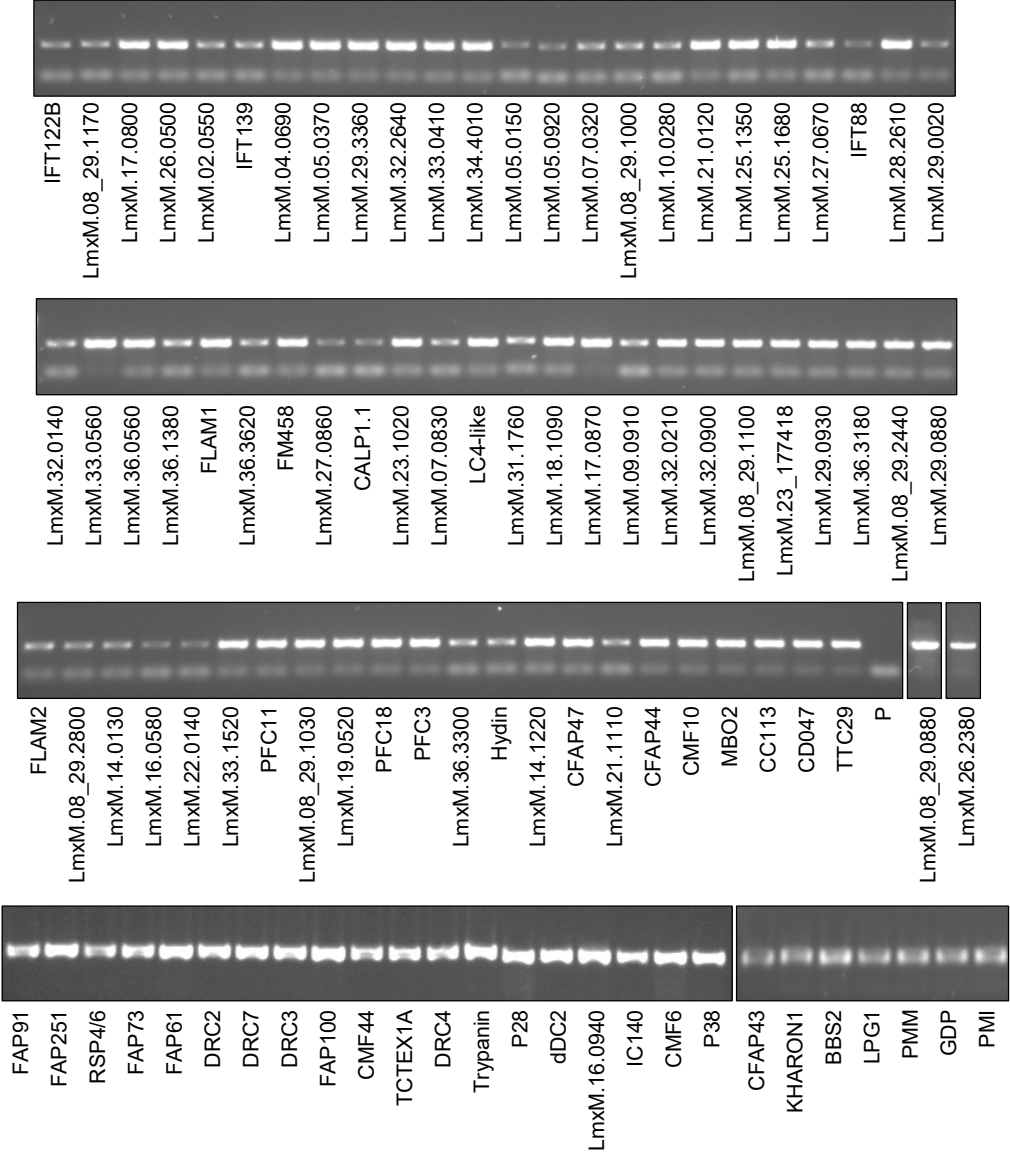

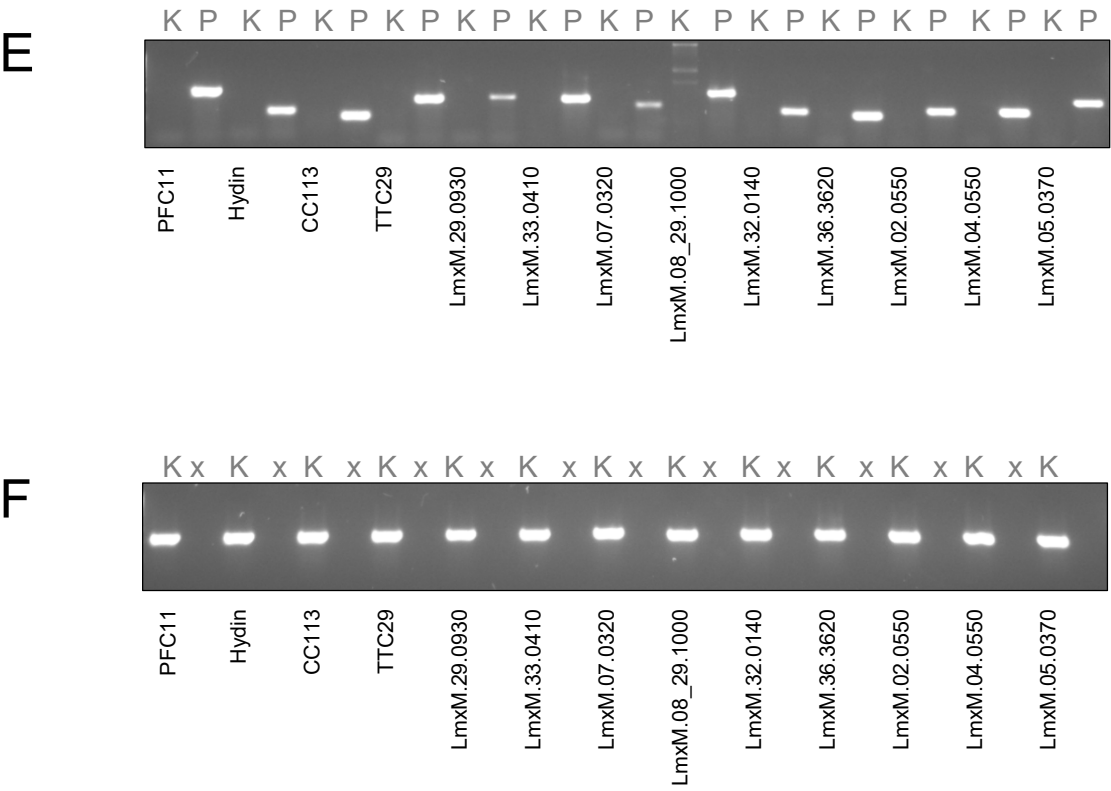

S8 Figure

Supplement: S8 Fig — Cartoons showing PCR strategy: (A) amplification of a fragment of the target gene ORF, (B) amplification of a fragment of the inserted blasticidin resistance gene (BlastR). (C) PCR products from target gene ORF run on agarose gel. Each panel shows the product obtained from the putative knockout cell line (K) and the parental L. mex Cas9 T7 cell line (P). The GeneIDs / gene names indicate the target gene. Absence of the target ORF PCR product was confirmed for all genes, except for LmxM.34.4010, CALP1.1, LmxM.09.0910 and CMF6. See S7 Table for amplicon sizes. Fainter bands below the target gene amplicon are likely primer dimers. (D) Presence of the BlastR PCR product was confirmed for all transfected cell lines; no BlastR PCR product was amplified from the parental genome (P). (E) The DNA of cell lines that yielded fainter bands of size considered too large to be primer dimers was probed again with a second primer pair and optimized PCR conditions using FastGene Optima polymerase (NIPPON Genetics Europe) according to the manufacture instructions. (F) Amplification of a fragment of the inserted blasticidin resistance gene (BlastR) using FastGene Optima polymerase. Empty lanes are marked with x. (PDF) [file ppat.1007828.s018.pdf]

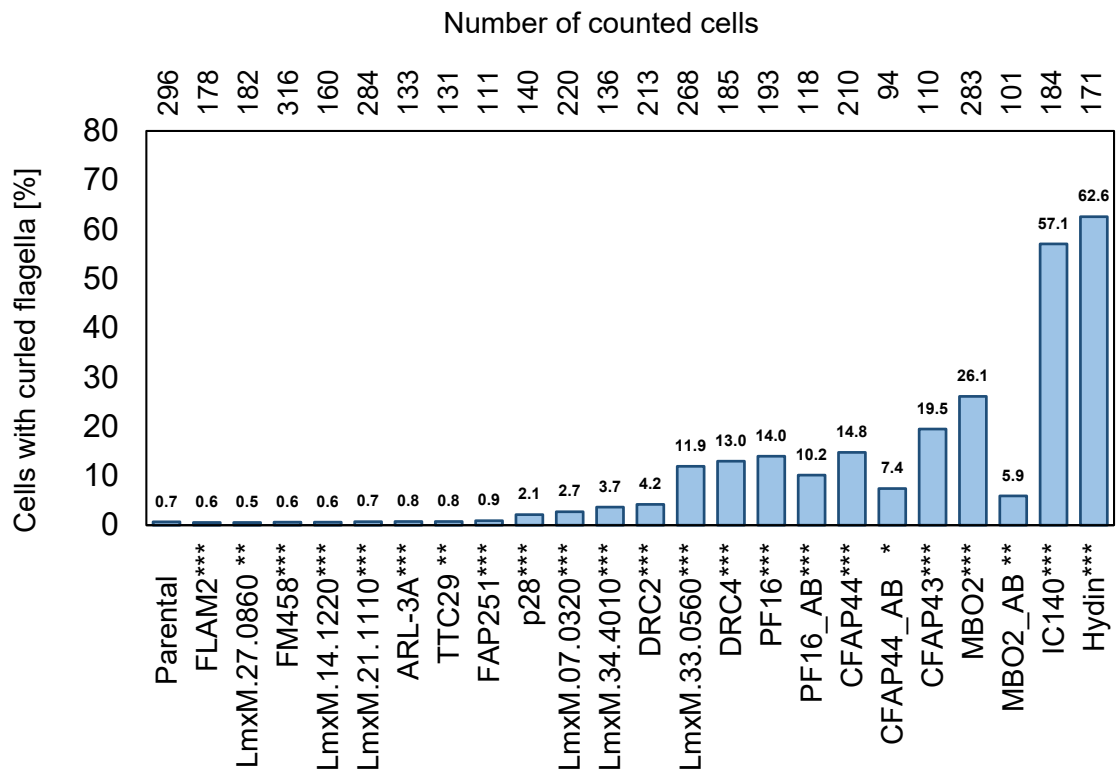

S9 Figure

Supplement: S9 Fig — Histogram showing the proportion of cells with curled flagella in the parental L. mex Cas9 T7 cell line, 20 different KO mutants and three add-back cell lines (AB). The GeneIDs / gene names indicate the target gene. Numbers above the bars indicate percentage of cells with curly flagella. Asterisks indicate motility phenotypes (see Fig 3). (PDF) [file ppat.1007828.s019.pdf]

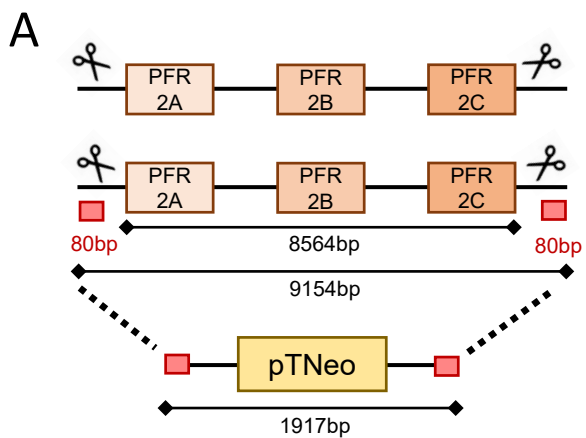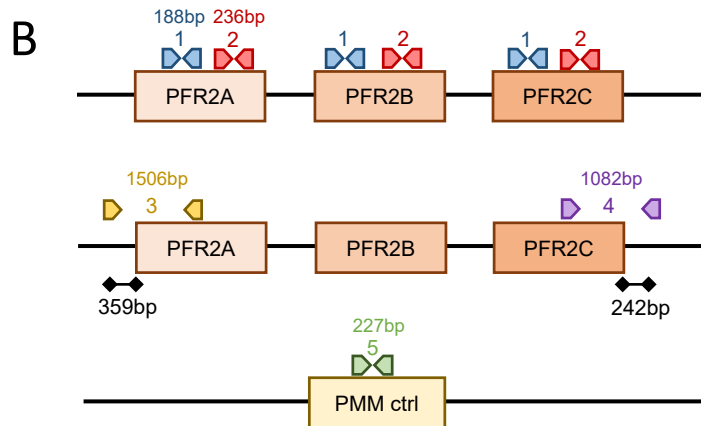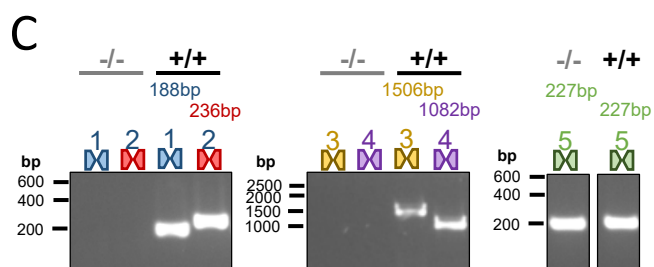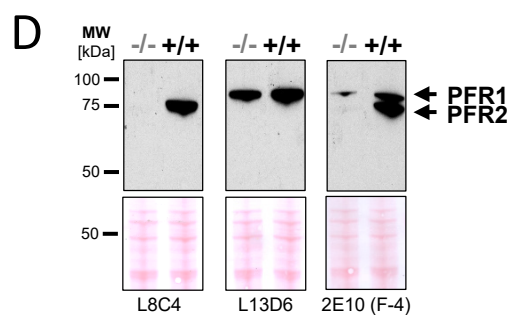

S10 Figure

Supplement: S10 Fig — (A) Strategy for deletion of PFR2 array, using CRISPR-Cas9 to insert a pTNeo cassette with homology arms flanking the array. (B) Cartoon showing location of five primer pairs used to validate KO cell line. (C) Results of diagnostic PCR. Primer pairs 1, 2, 3 and 4 specific to PFR2 array sequences only yield a product in the parental cell line (+/+) but not in the KO line (-/-). Primer pair 5, specific to an unrelated gene (PMM, LmxM.36.1960) yields a product in the parental and the KO cell line. (A), (B) and (C) Size of deleted PFR2 array and expected PCR amplicons are indicated. (D) western blots of parental and PFR2 KO protein lysates. Upper panel: blots probed with three different monoclonal antibodies: L8C4 [103] (1:1000 dilution in TBST 1% skim milk powder, sigma) is specific to PFR2, L13D6 [103] (1:20 dilution in TBST 1% skim milk powder) detects PFR1 and 2E10 detects both PFR1 and PFR2 [104] (1:1000 dilution in TBST 5% skim milk powder). No PFR2 signal is detected in the PFR2 KO line. Lower panel: Ponceau red stained membrane. (PDF) [file ppat.1007828.s020.pdf]

3 replicates

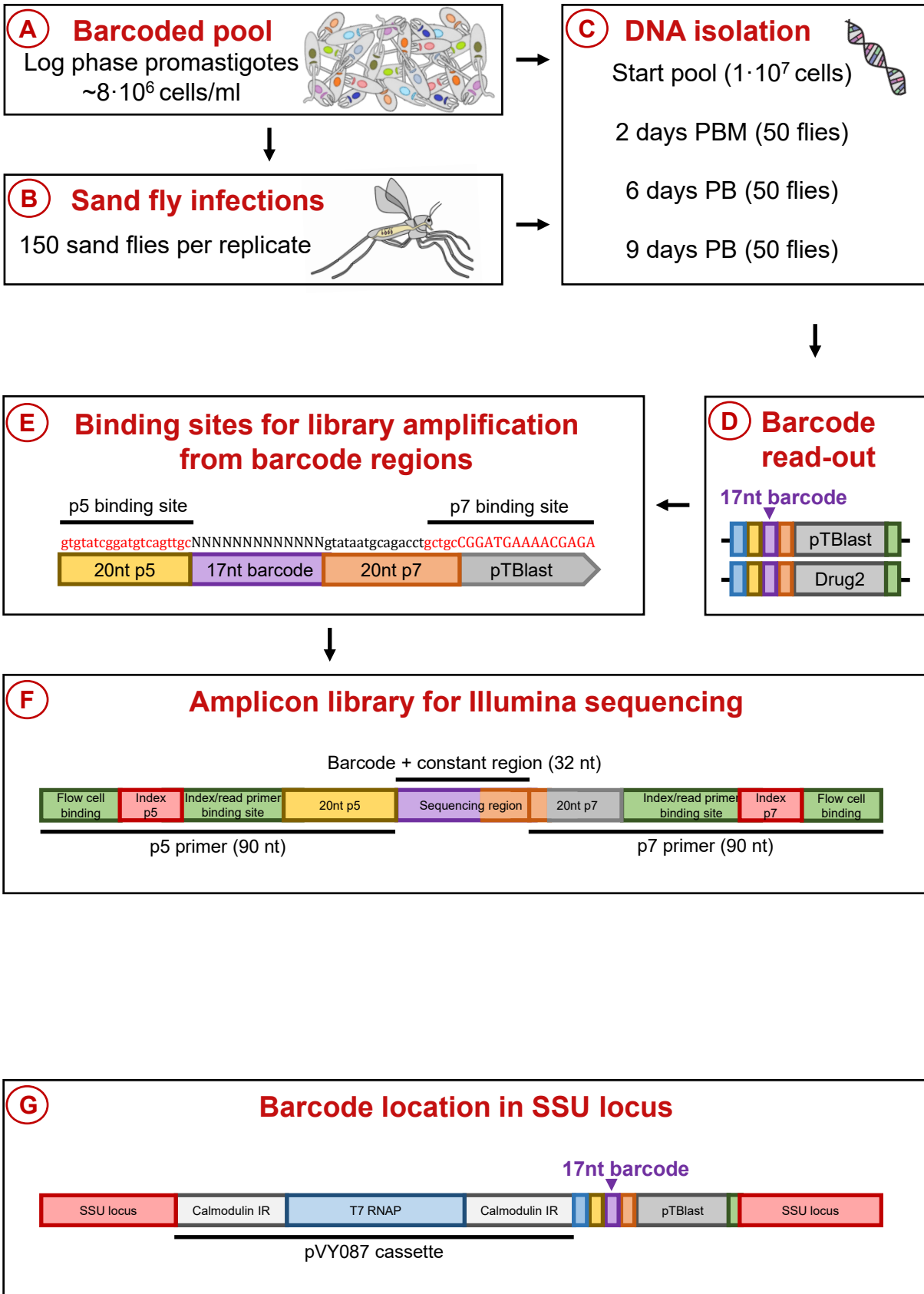

Supplement: S11 Fig — (A) Barcoded cell lines in log phase of growth were pooled in equal proportion. (B) For each replicate, 150 sand flies were fed on rabbit blood containing the pooled Leishmania. (C) DNA was isolated from the pooled population before feeding the sand flies. Two, six and nine days post blood meal, DNA was isolated from 50 infected flies. (D, E) The 17-nt unique barcode and adjacent constant region was amplified from the isolated DNA. Primer binding sites for indexed p5 and p7 Illumina sequencing primers are indicated in red. (F) Final amplicon library constructs subjected to Illumina sequencing. Samples were multiplexed using different p5 and p7 indices. (G) Diagram showing location of the barcode in control cell lines. The 17-nt barcode, linked to a blasticidin resistance gene, replaces the SatR gene in the pVY087 cassette [105] in L. mex Cas9 T7 cell lines [23]. (PDF) [file ppat.1007828.s021.pdf]

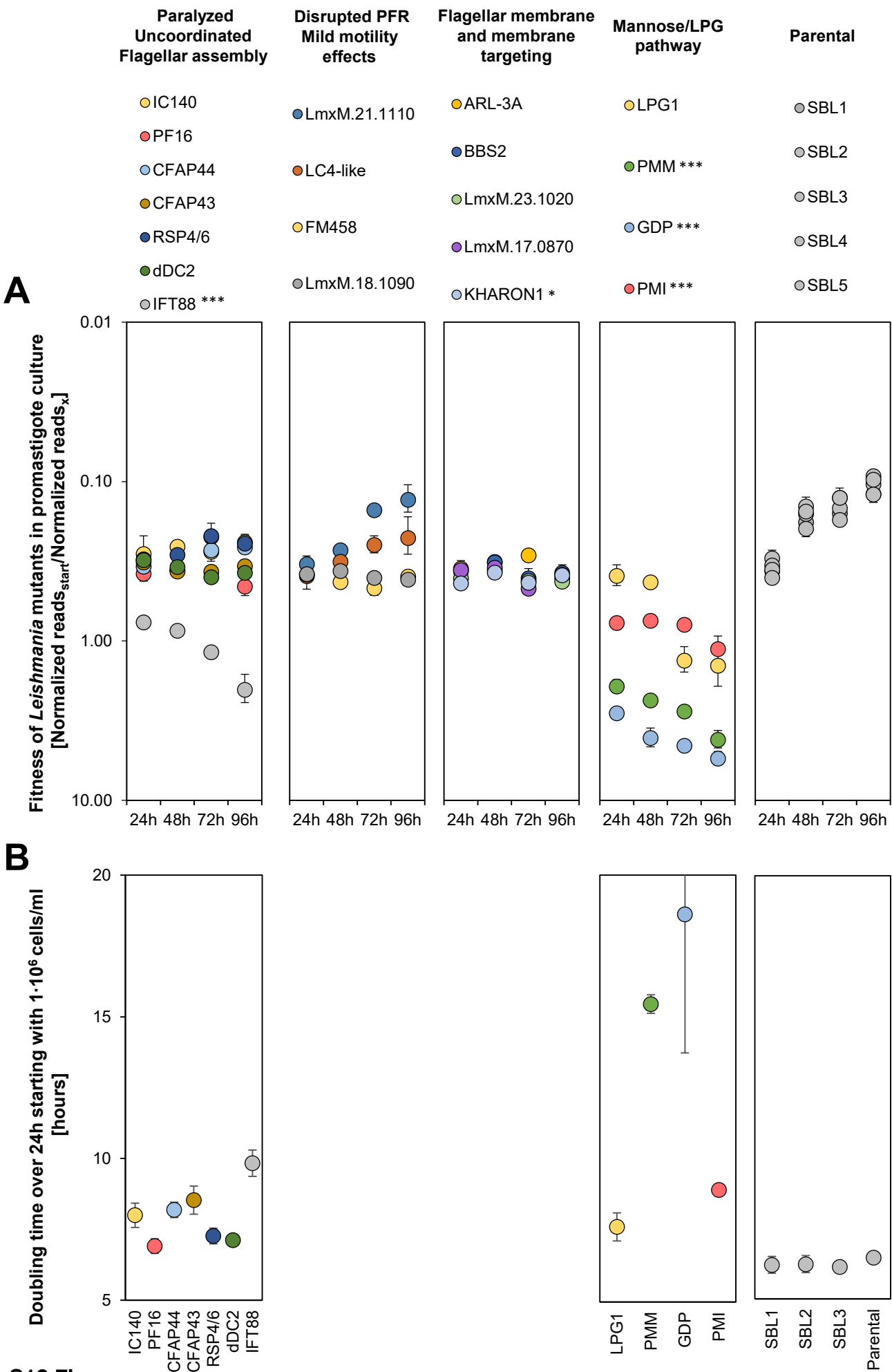

Supplement: S12 Fig — (A) Relative growth rates of promastigotes in mixed populations in culture. Barcoded cell lines in log phase of growth were pooled in equal proportion to a density of 1·106 cells/ml and DNA was extracted at the indicated time points. The plots display abundance of barcodes at the indicted time points relative to the abundance of this barcode in the initial pool. Mutants are grouped according to the predicted functions of the deleted genes and severity of the phenotype in the sand flies, as shown in Fig 6. Data points represent the average of three replicates. Error bars show the standard deviation of the mean of the three replicates. For the 24h time point, measurements were compared (two-sided t-test) to the average of all five parental controls (p-values are indicated: *≤0.05, **≤0.005, ***≤0.0005). (B) Doubling times measured for individual promastigote cell lines in culture. Cells were seeded at 1·106 cells/ml and density was measured after 24h. Doubling time was calculated by (24/(log2(t2/ t1))). Data points represent the average of four replicates, except for ΔPMM and ΔGDP-MP, which were measured twice. Error bars show the standard deviation of the mean. (PDF) [file ppat.1007828.s022.pdf]

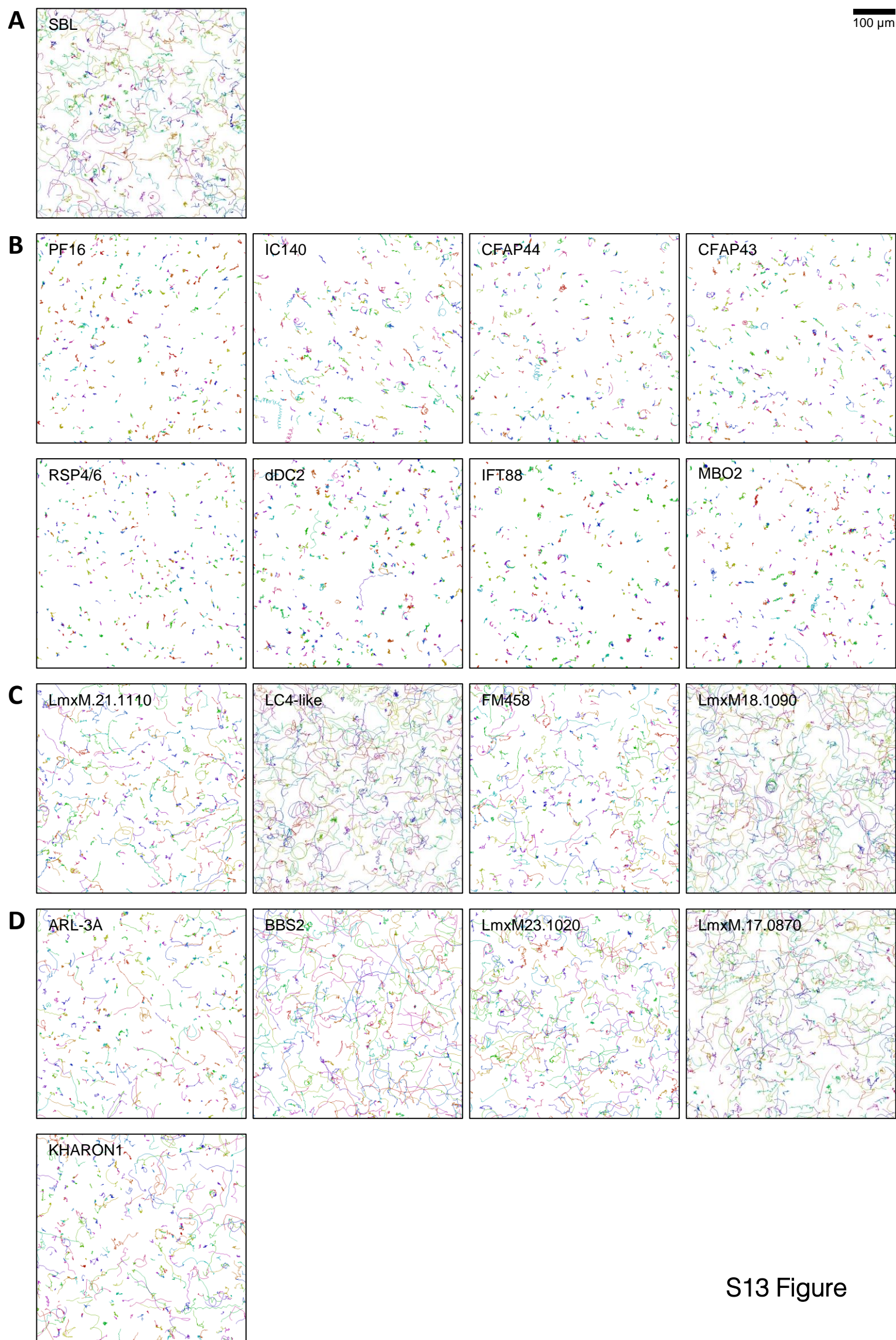

S13 Figure

Supplement: S13 Fig — Swimming paths extracted from darkfield microscopy timelapse videos are shown for cell lines used in sand fly infections (A) control cell line (SBL, barcoded L. mex Cas9 T7), (B) gene deletions resulting in paralysis or uncoordinated swimming, (C) gene deletions with mild motility defects, (D) gene deletions targeting flagellar membrane proteins and protein trafficking. 400 tracks are shown for each cell line. (PDF) [file ppat.1007828.s023.pdf]
