# Supplementary material for: Genetic dissection of a Leishmania flagellar proteome demonstrates requirement for directional motility in sand fly infections
Source: PLoS Pathog. 2019 Jun 26;15(6):e1007828. doi: 10.1371/journal.ppat.1007828 (PMC6615630; doi:10.1371/journal.ppat.1007828)
Supplement: S4 Fig — (A) Pearson’s correlation coefficient and (B) Spearman’s rank of spectral indices for 1918 detected proteins (overlap of both biological replicates) of all cell fractions. (PDF) [file ppat.1007828.s014.pdf]

Pearson correlation coefficient

A

	C_l 2	C_l 1	C_S 2	C_S 1	F_l 2	F_l 1	F_S 2	F_S 1
**C_l 2**	1.00	0.72	0.28	0.27	0.68	0.49	0.16	0.21
**C_l 1**	0.72	1.00	0.53	0.62	0.53	0.56	0.28	0.38
**C_S 2**	0.28	0.53	1.00	0.78	0.58	0.44	0.58	0.61
**C_S 1**	0.27	0.62	0.78	1.00	0.41	0.45	0.50	0.60
**F_l 2**	0.68	0.53	0.58	0.41	1.00	0.73	0.65	0.57
**F_l 1**	0.49	0.56	0.44	0.45	0.73	1.00	0.40	0.50
**F_S 2**	0.16	0.28	0.58	0.50	0.65	0.40	1.00	0.81
**F_S 1**	0.21	0.38	0.61	0.60	0.57	0.50	0.81	1.00

Spearman's rank

B

	C_l 2	C_l 1	C_S 2	C_S 1	F_l 2	F_l 1	F_S 2	F_S 1
**C_l 2**	1.00	0.86	0.62	0.58	0.51	0.46	0.49	0.54
**C_l 1**	0.86	1.00	0.57	0.61	0.47	0.48	0.44	0.55
**C_S 2**	0.62	0.57	1.00	0.88	0.36	0.32	0.72	0.74
**C_S 1**	0.58	0.61	0.88	1.00	0.29	0.33	0.67	0.75
**F_l 2**	0.51	0.47	0.36	0.29	1.00	0.84	0.47	0.42
**F_l 1**	0.46	0.48	0.32	0.33	0.84	1.00	0.43	0.49
**F_S 2**	0.49	0.44	0.72	0.67	0.47	0.43	1.00	0.83
**F_S 1**	0.54	0.55	0.74	0.75	0.42	0.49	0.83	1.00

S4 Figure
