# Supplementary material for: Genetic dissection of a Leishmania flagellar proteome demonstrates requirement for directional motility in sand fly infections
Source: PLoS Pathog. 2019 Jun 26;15(6):e1007828. doi: 10.1371/journal.ppat.1007828 (PMC6615630; doi:10.1371/journal.ppat.1007828)
Supplement: S7 Fig — Widefield epifluorescence micrographs of L. mexicana cells. Left image: merged phase-contrast (grey), red (Hoechst-stained DNA), and green (mNG or eYFP) channels. Right image: greyscale image of green channel (mNG or eYFP signal). For each tagged protein, the relevant parental cell line (wild type for proteins tagged with eYFP, L. mex Cas9 T7 for proteins tagged with mNG) was imaged, using the same acquisition settings and image processing parameters as for the tagged cell line. For each protein, panels on the left show N-terminal tags, panel on the right C-terminal tags. A choice was made to tag only the C-terminus for proteins with a predicted signal peptide or an N-Myristoylation motif. Asterisks next to the GeneIDs indicate that the corresponding knockout cell lines showed a motility phenotype (see Fig 3); A # indicates that the corresponding knockout cell line was passaged through sand flies (see Figs 6 and 7). (PDF) [file ppat.1007828.s017.pdf]

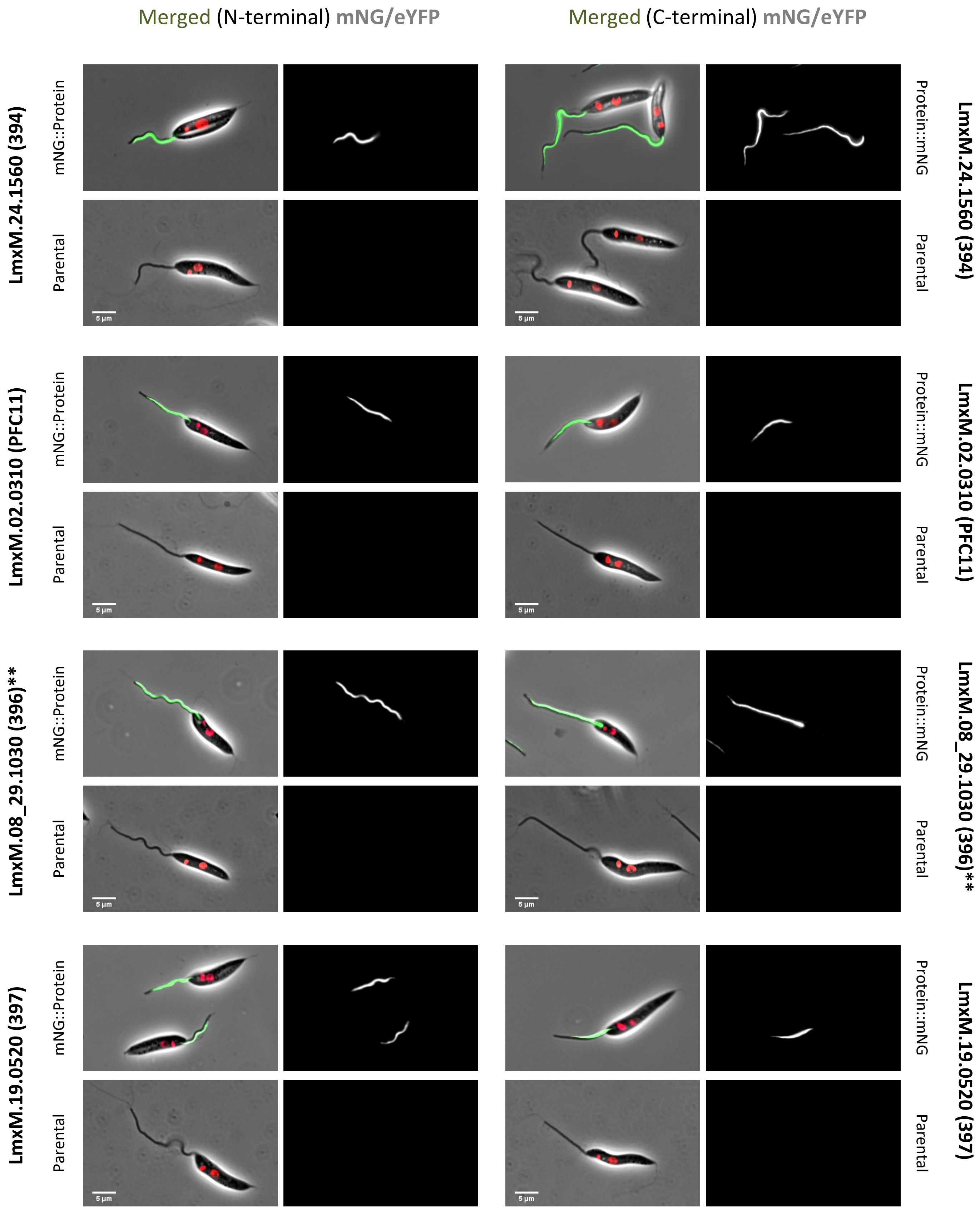
Merged (N-terminal) mNG/eYFP
Merged (C-terminal) mNG/eYFP
LmxM.24.1560 (394)
mNG::Protein
Parental
Protein::mNG
LmxM.02.0310 (PFC11)
LmxM.08_29.1030 (396)**
LmxM.19.0520 (397)
5 µm

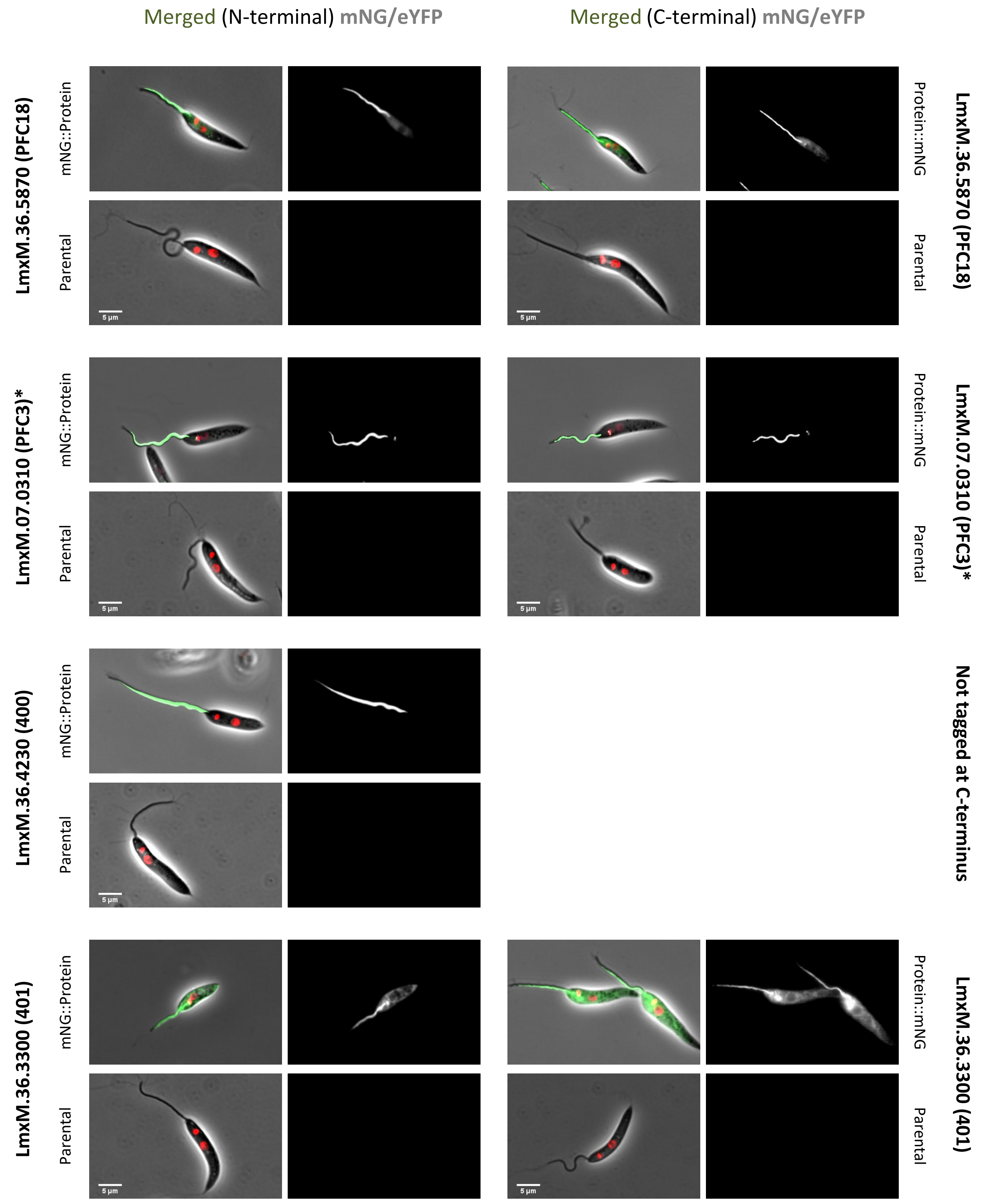

Merged (N-terminal) mNG/eYFP
Merged (C-terminal) mNG/eYFP
LmxM.36.5870 (PFC18)
mNG::Protein
Parental
Protein::mNG
LmxM.07.0310 (PFC3)*
LmxM.36.4230 (400)
Not tagged at C-terminus
LmxM.36.3300 (401)
5 µm

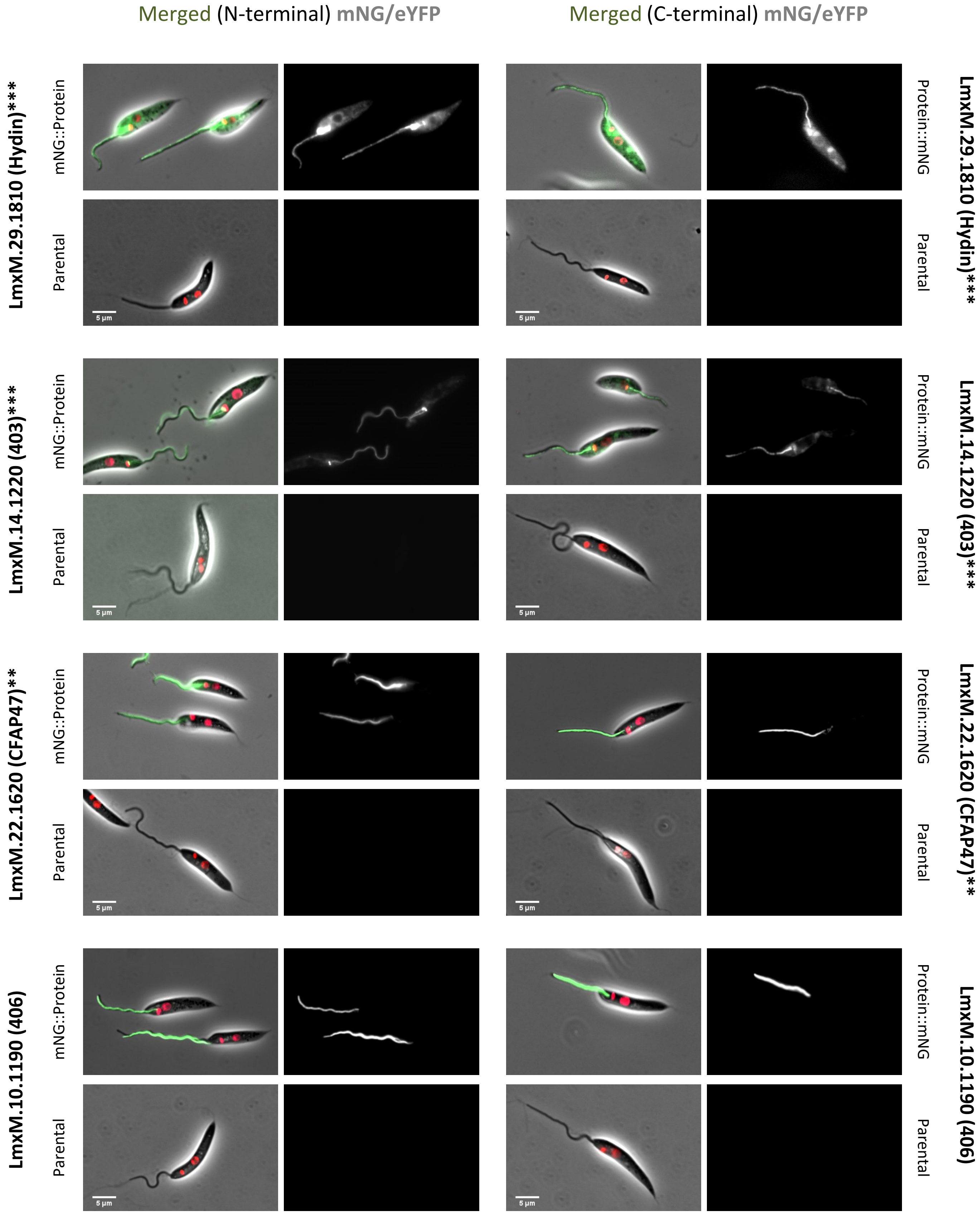

Merged (N-terminal) mNG/eYFP
Merged (C-terminal) mNG/eYFP
LmxM.29.1810 (Hydin)***
mNG::Protein
Parental
5 µm
Protein::mNG
Parental
LmxM.29.1810 (Hydin)***
LmxM.14.1220 (403)***
mNG::Protein
Parental
5 µm
Protein::mNG
Parental
LmxM.14.1220 (403)***
LmxM.22.1620 (CFAP47)**
mNG::Protein
Parental
5 µm
Protein::mNG
Parental
LmxM.22.1620 (CFAP47)**
LmxM.10.1190 (406)
mNG::Protein
Parental
5 µm
Protein::mNG
Parental
LmxM.10.1190 (406)

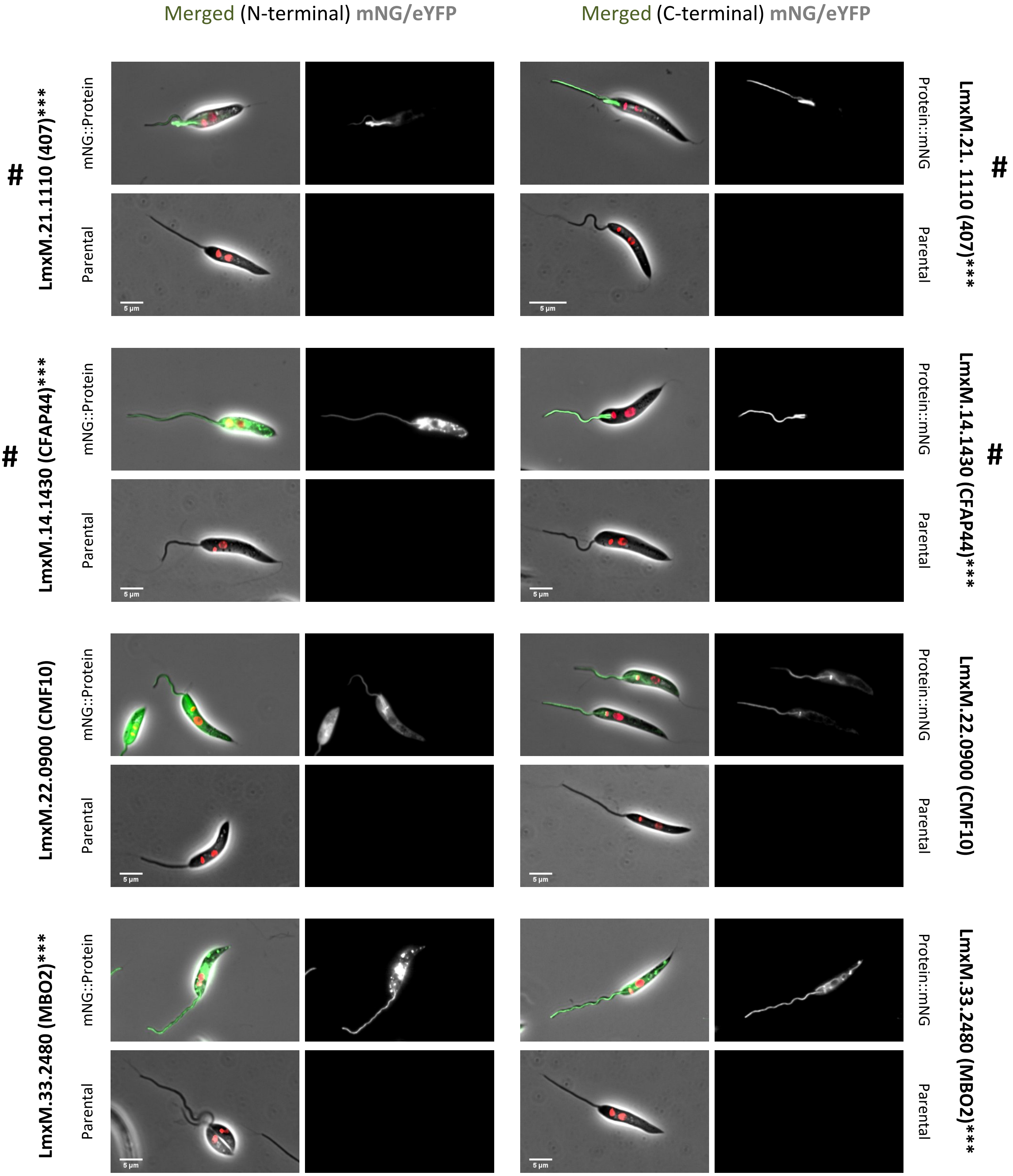

Merged (N-terminal) mNG/eYFP
Merged (C-terminal) mNG/eYFP
#
LmxM.21.1110 (407)***
mNG::Protein
Parental
5 µm
Protein::mNG
Parental
LmxM.21. 1110 (407)***
#
#
LmxM.14.1430 (CFAP44)***
mNG::Protein
Parental
Protein::mNG
Parental
LmxM.14.1430 (CFAP44)***
#
LmxM.22.0900 (CMF10)
mNG::Protein
Parental
Protein::mNG
Parental
LmxM.22.0900 (CMF10)
LmxM.33.2480 (MBO2)***
mNG::Protein
Parental
Protein::mNG
Parental
LmxM.33.2480 (MBO2)***

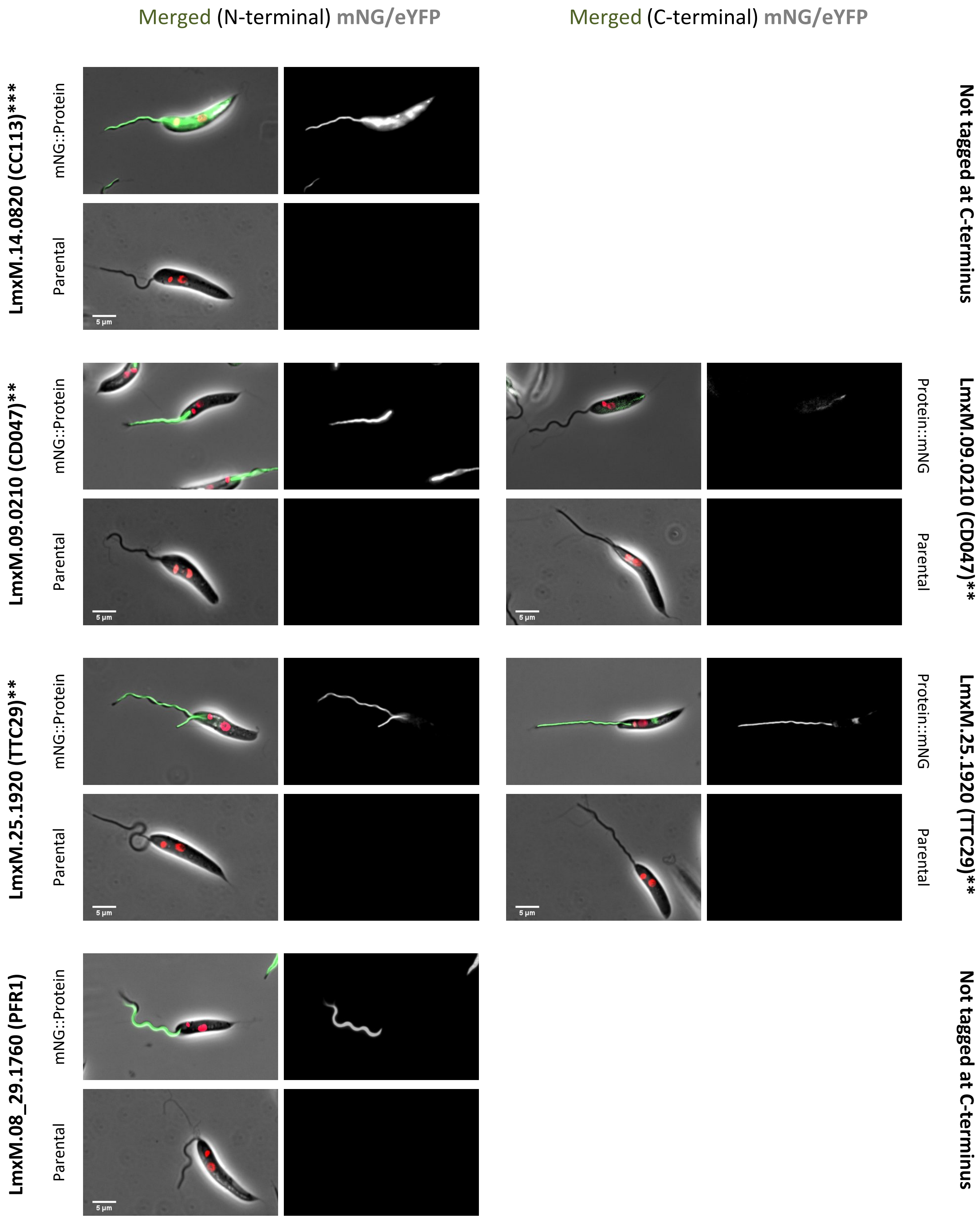

Merged (N-terminal) mNG/eYFP
Merged (C-terminal) mNG/eYFP
LmxM.14.0820 (CC113)***
mNG::Protein
Parental
5 µm
Not tagged at C-terminus
LmxM.09.0210 (CD047)**
mNG::Protein
Parental
5 µm
Protein::mNG
Parental
LmxM.09.0210 (CD047)**
LmxM.25.1920 (TTC29)**
mNG::Protein
Parental
5 µm
Protein::mNG
Parental
LmxM.25.1920 (TTC29)**
LmxM.08_29.1760 (PFR1)
mNG::Protein
Parental
5 µm
Not tagged at C-terminus

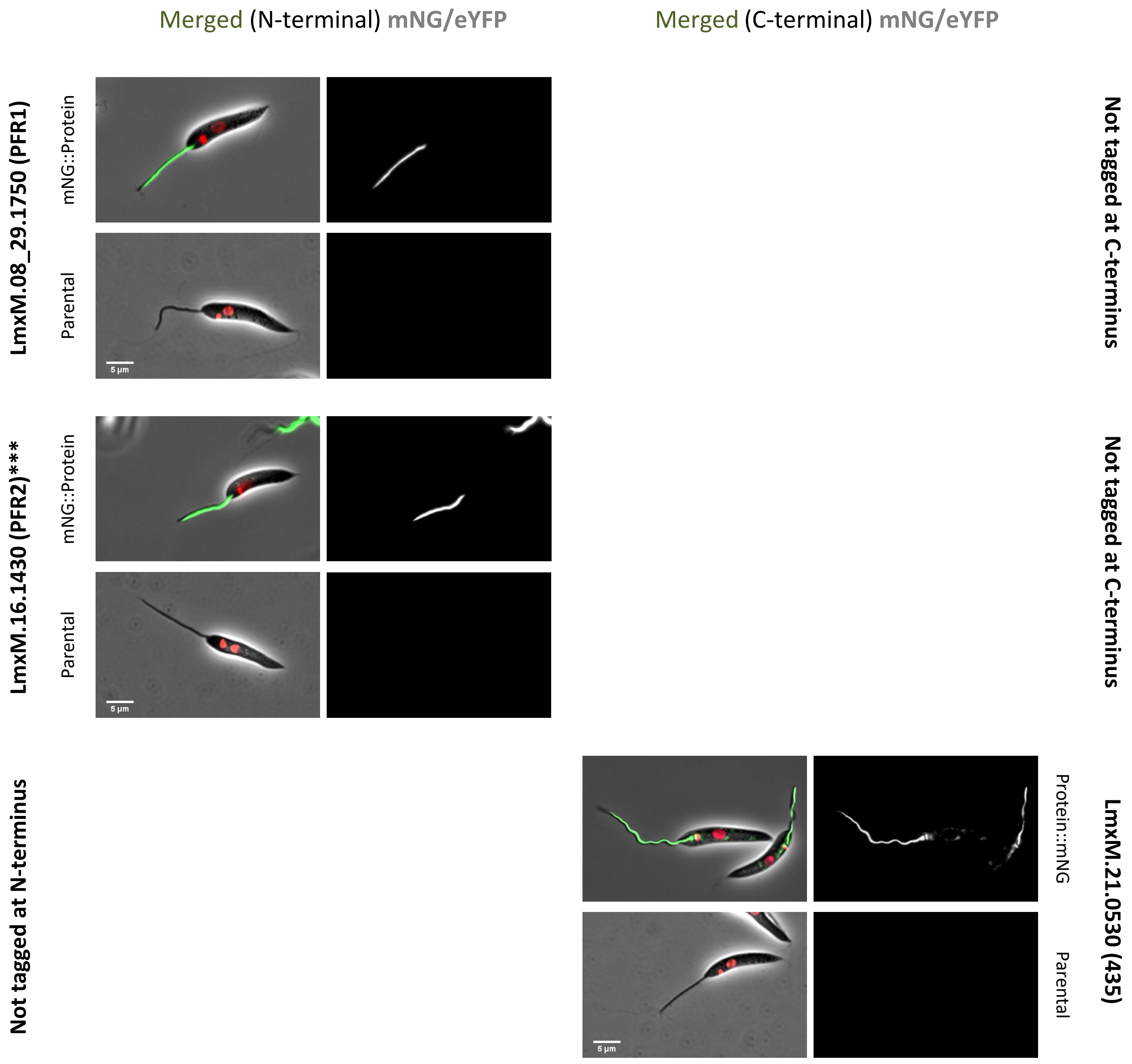
Merged (N-terminal) mNG/eYFP
Merged (C-terminal) mNG/eYFP
LmxM.08_29.1750 (PFR1)
mNG::Protein
Parental
5 µm
Not tagged at C-terminus
LmxM.16.1430 (PFR2)***
mNG::Protein
Parental
5 µm
Not tagged at C-terminus
Not tagged at N-terminus
LmxM.21.0530 (435)
Protein::mNG
Parental
5 µm

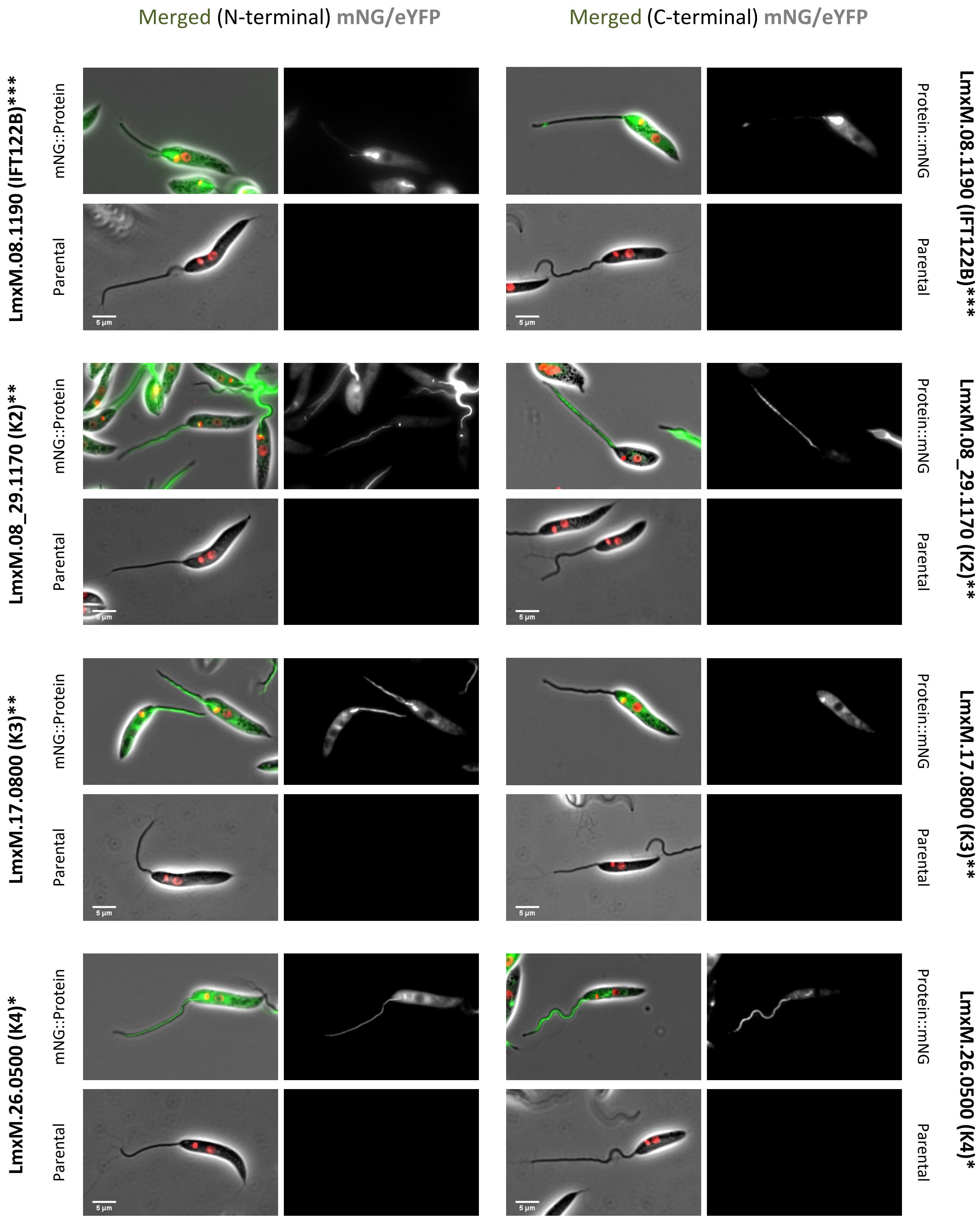

Merged (N-terminal) mNG/eYFP
Merged (C-terminal) mNG/eYFP
LmxM.08.1190 (IFT122B)***
LmxM.08_29.1170 (K2)**
LmxM.17.0800 (K3)**
LmxM.26.0500 (K4)*
mNG::Protein
Protein::mNG
Parental
5 µm

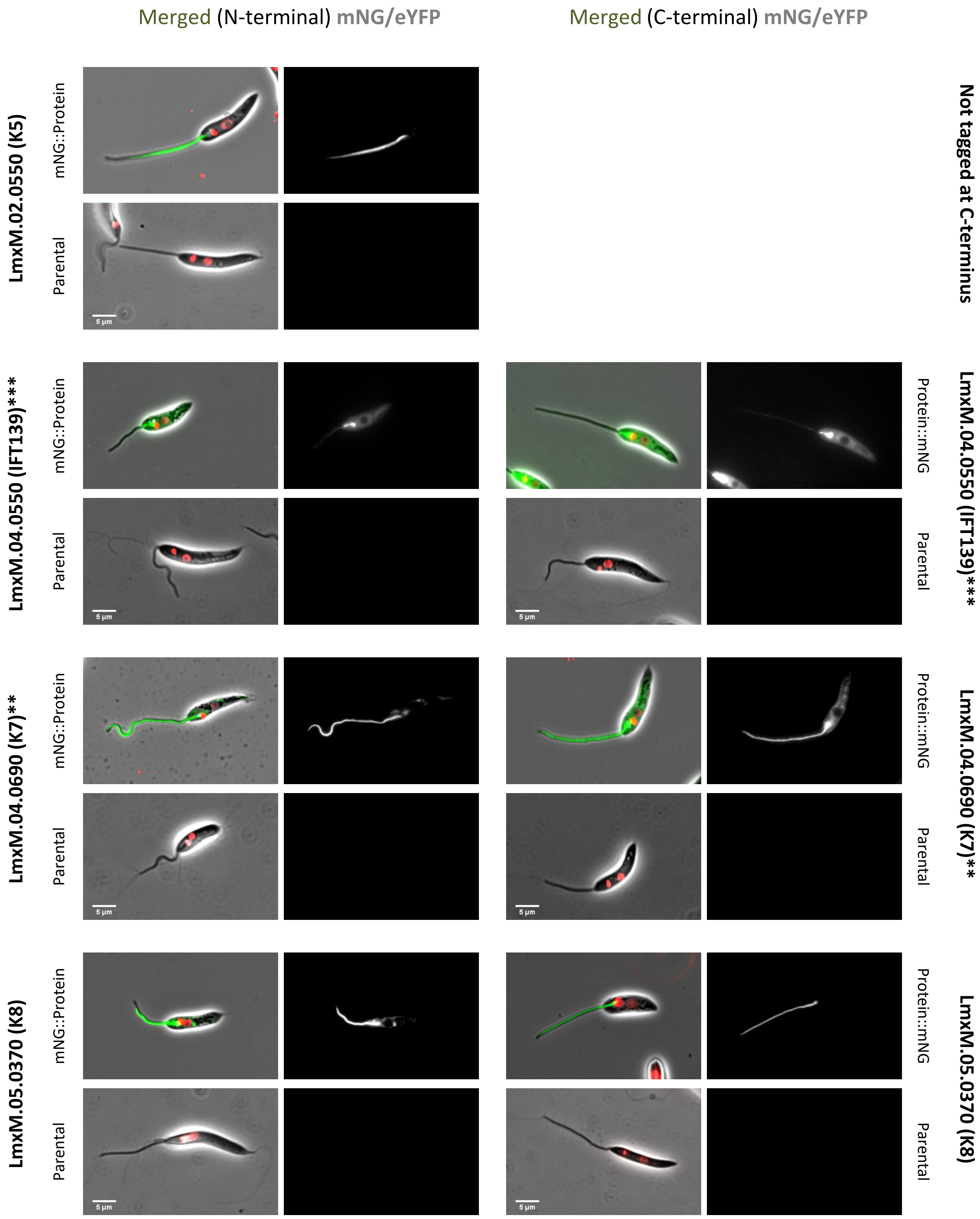

Merged (N-terminal) mNG/eYFP
Merged (C-terminal) mNG/eYFP
LmxM.02.0550 (K5)
mNG::Protein
Parental
Not tagged at C-terminus
LmxM.04.0550 (IFT139)***
Protein::mNG
LmxM.04.0550 (IFT139)***
LmxM.04.0690 (K7)**
LmxM.04.0690 (K7)**
LmxM.05.0370 (K8)
LmxM.05.0370 (K8)
5 µm

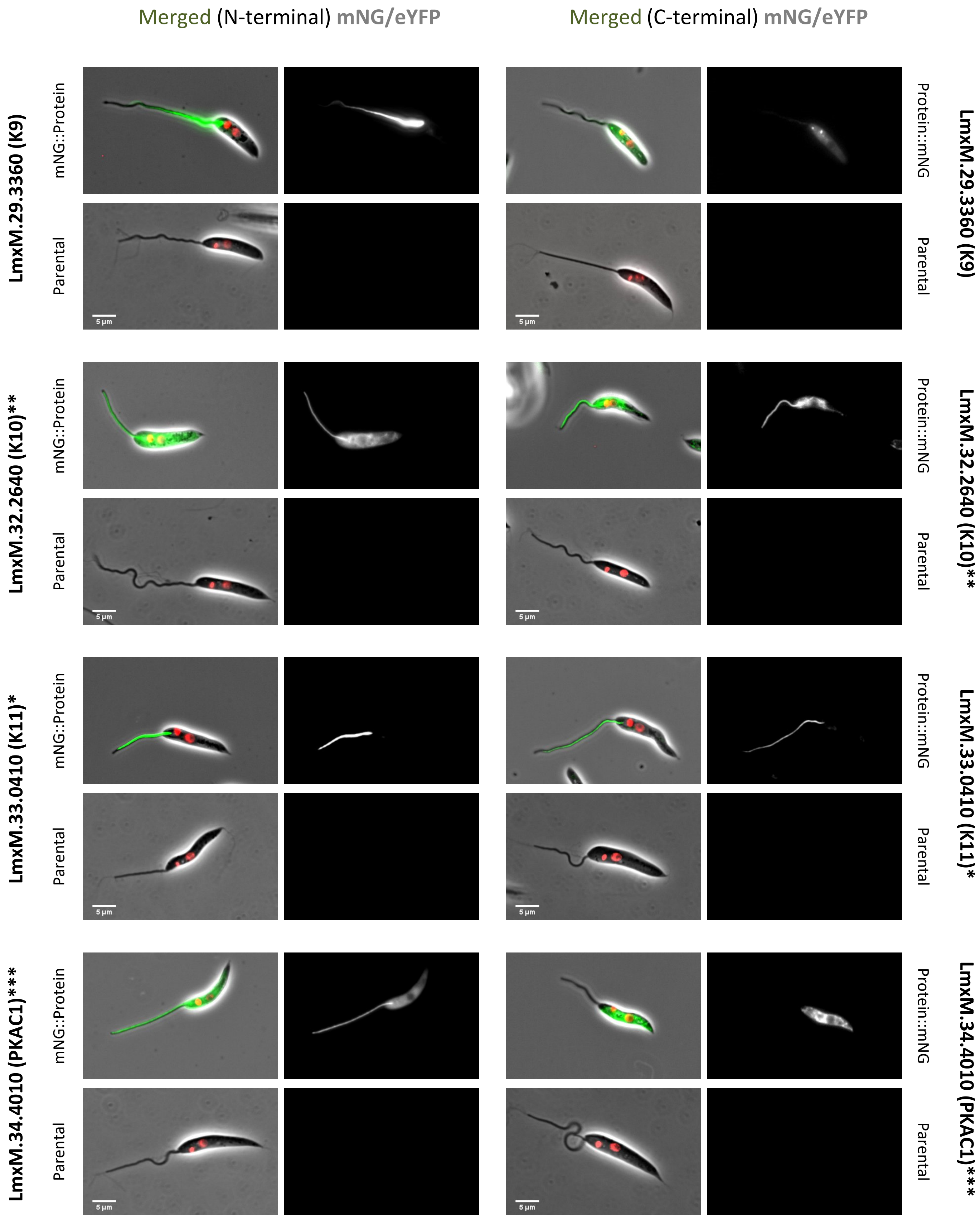

Merged (N-terminal) mNG/eYFP
Merged (C-terminal) mNG/eYFP
LmxM.29.3360 (K9)
mNG::Protein
Parental
Protein::mNG
Parental
LmxM.32.2640 (K10)**
LmxM.33.0410 (K11)*
LmxM.34.4010 (PKAC1)***
5 µm

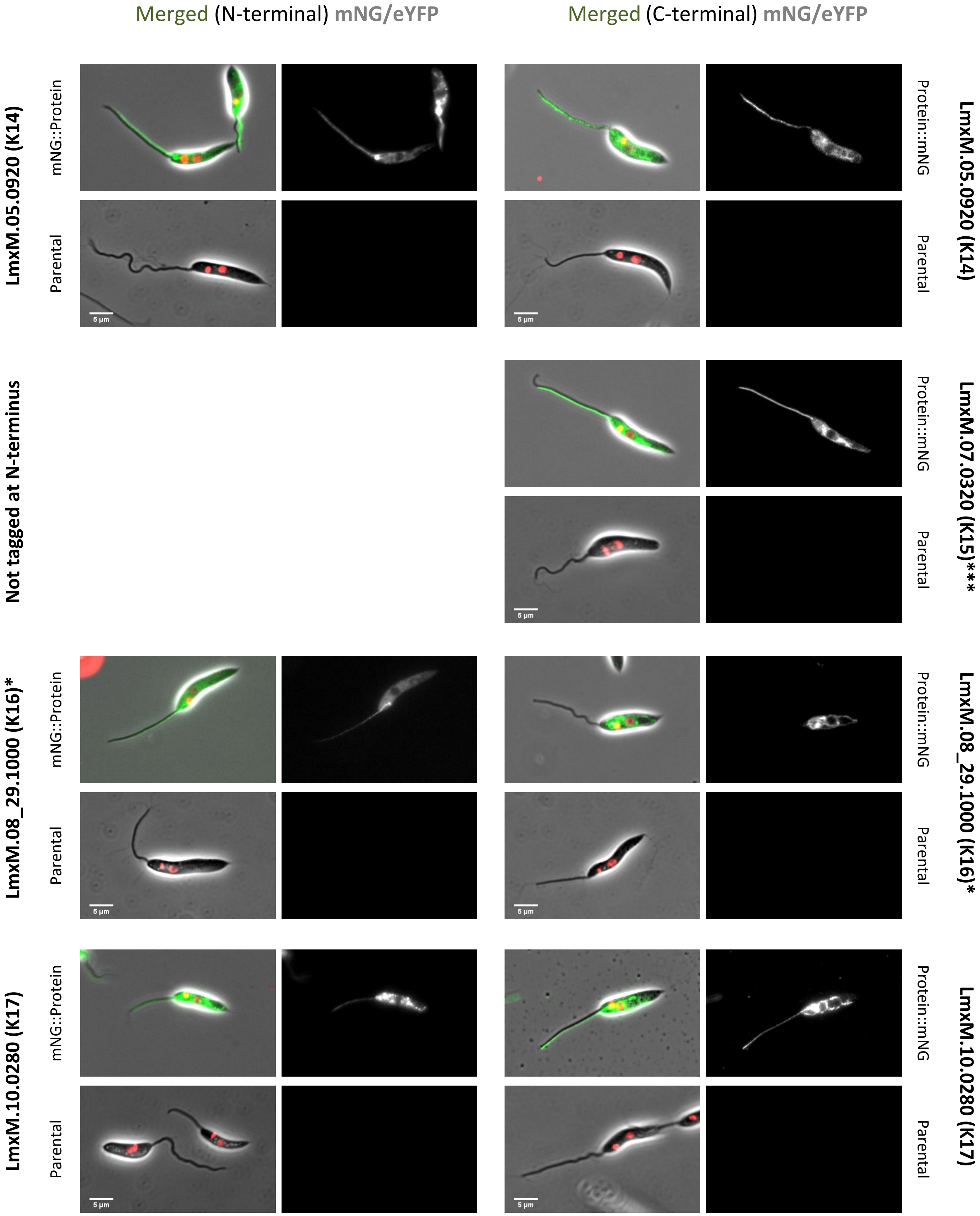
Merged (N-terminal) mNG/eYFP
Merged (C-terminal) mNG/eYFP
LmxM.05.0920 (K14)
mNG::Protein
Parental
Protein::mNG
Parental
LmxM.05.0920 (K14)
Not tagged at N-terminus
LmxM.07.0320 (K15)**
LmxM.08_29.1000 (K16)*
LmxM.08_29.1000 (K16)*
LmxM.10.0280 (K17)
LmxM.10.0280 (K17)
5 µm

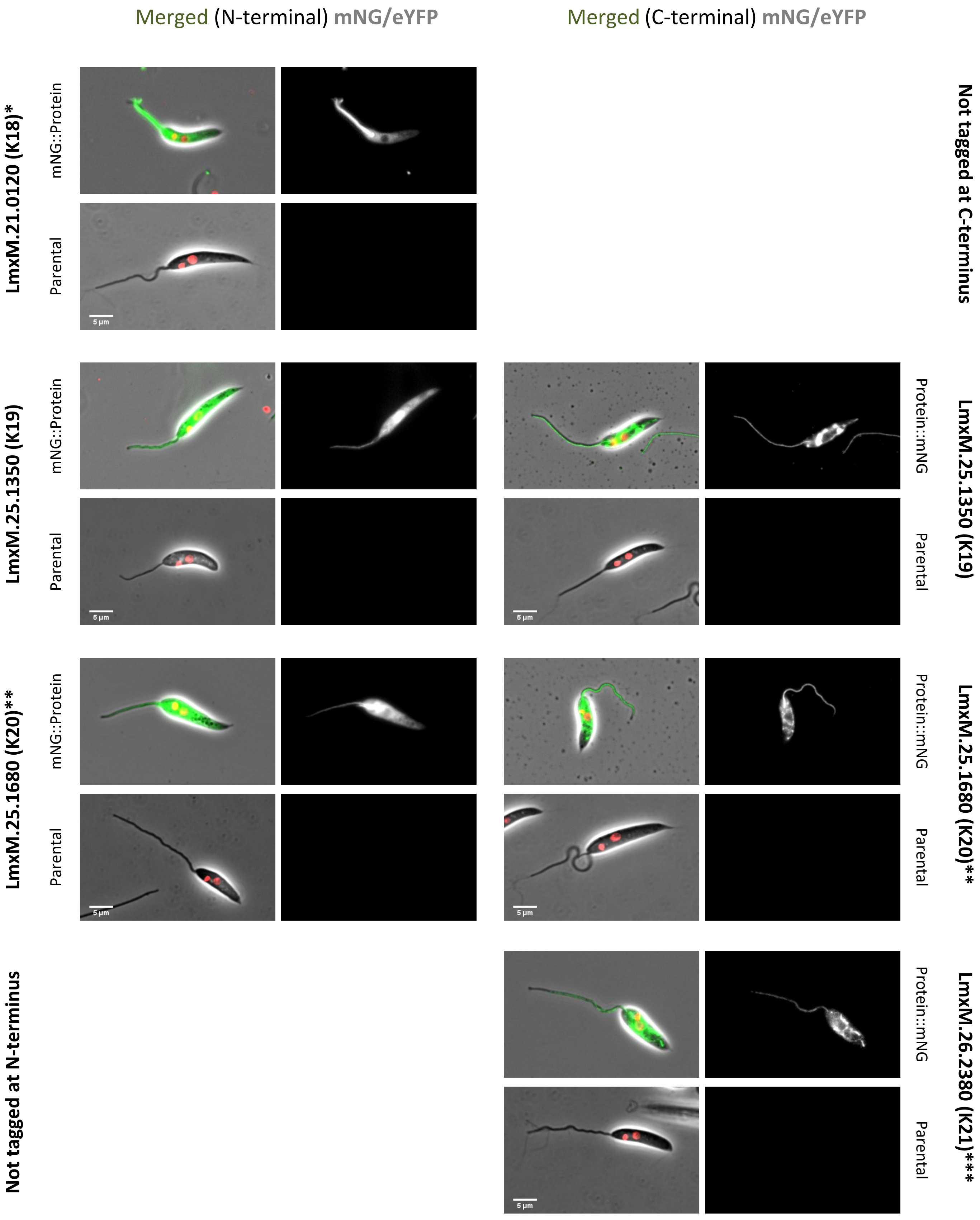

Merged (N-terminal) mNG/eYFP
Merged (C-terminal) mNG/eYFP
LmxM.21.0120 (K18)*
mNG::Protein
Parental
Not tagged at C-terminus
LmxM.25.1350 (K19)
mNG::Protein
Parental
Protein::mNG
Parental
LmxM.25.1350 (K19)
LmxM.25.1680 (K20)**
mNG::Protein
Parental
Protein::mNG
Parental
LmxM.25.1680 (K20)**
Not tagged at N-terminus
Protein::mNG
Parental
LmxM.26.2380 (K21)**
5 µm

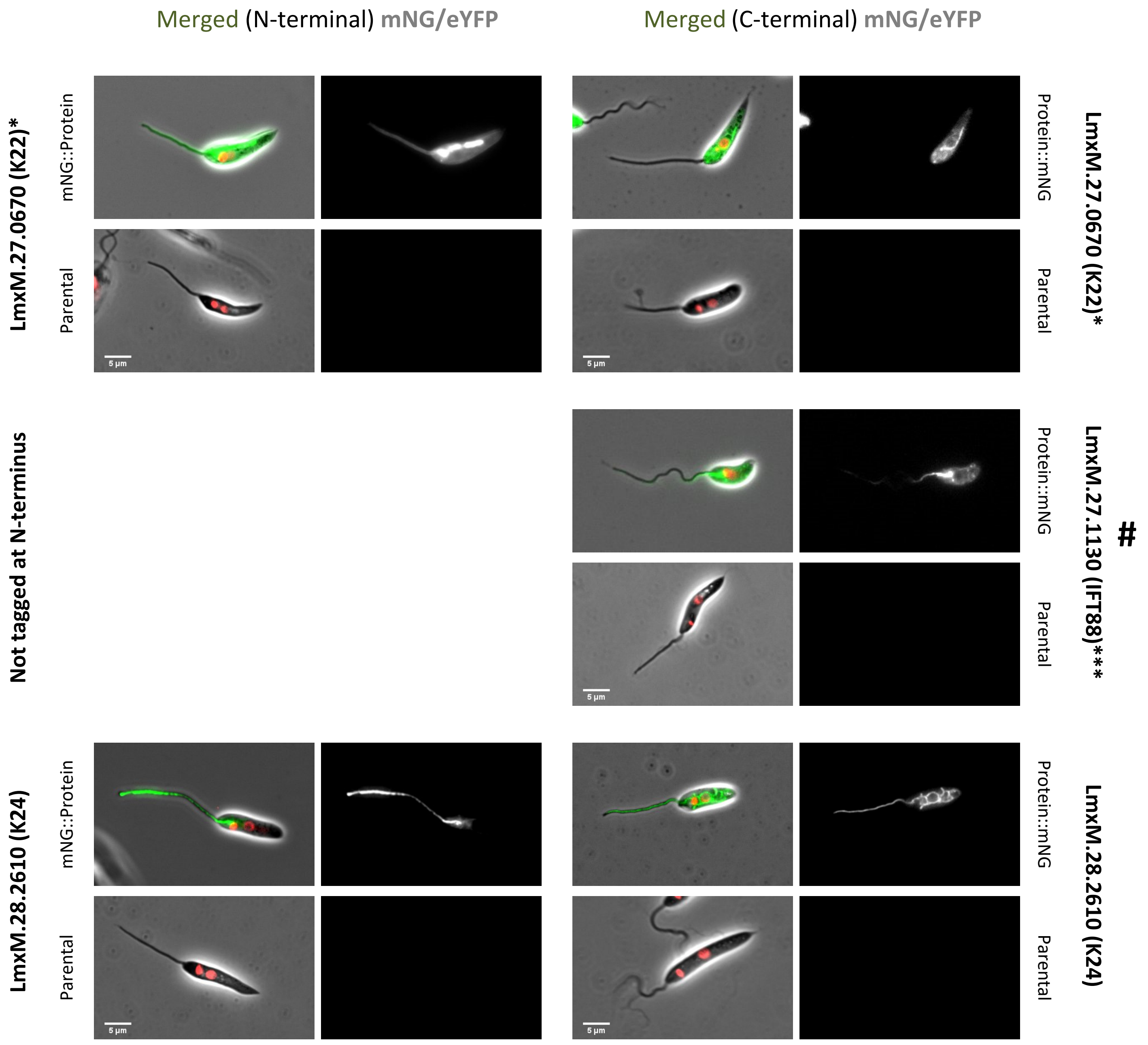

Merged (N-terminal) mNG/eYFP
Merged (C-terminal) mNG/eYFP
LmxM.27.0670 (K22)*
mNG::Protein
Parental
Protein::mNG
Parental
LmxM.27.0670 (K22)*
5 µm
Not tagged at N-terminus
Protein::mNG
Parental
LmxM.27.1130 (IFT88)***
#
LmxM.28.2610 (K24)
mNG::Protein
Parental
Protein::mNG
Parental
LmxM.28.2610 (K24)

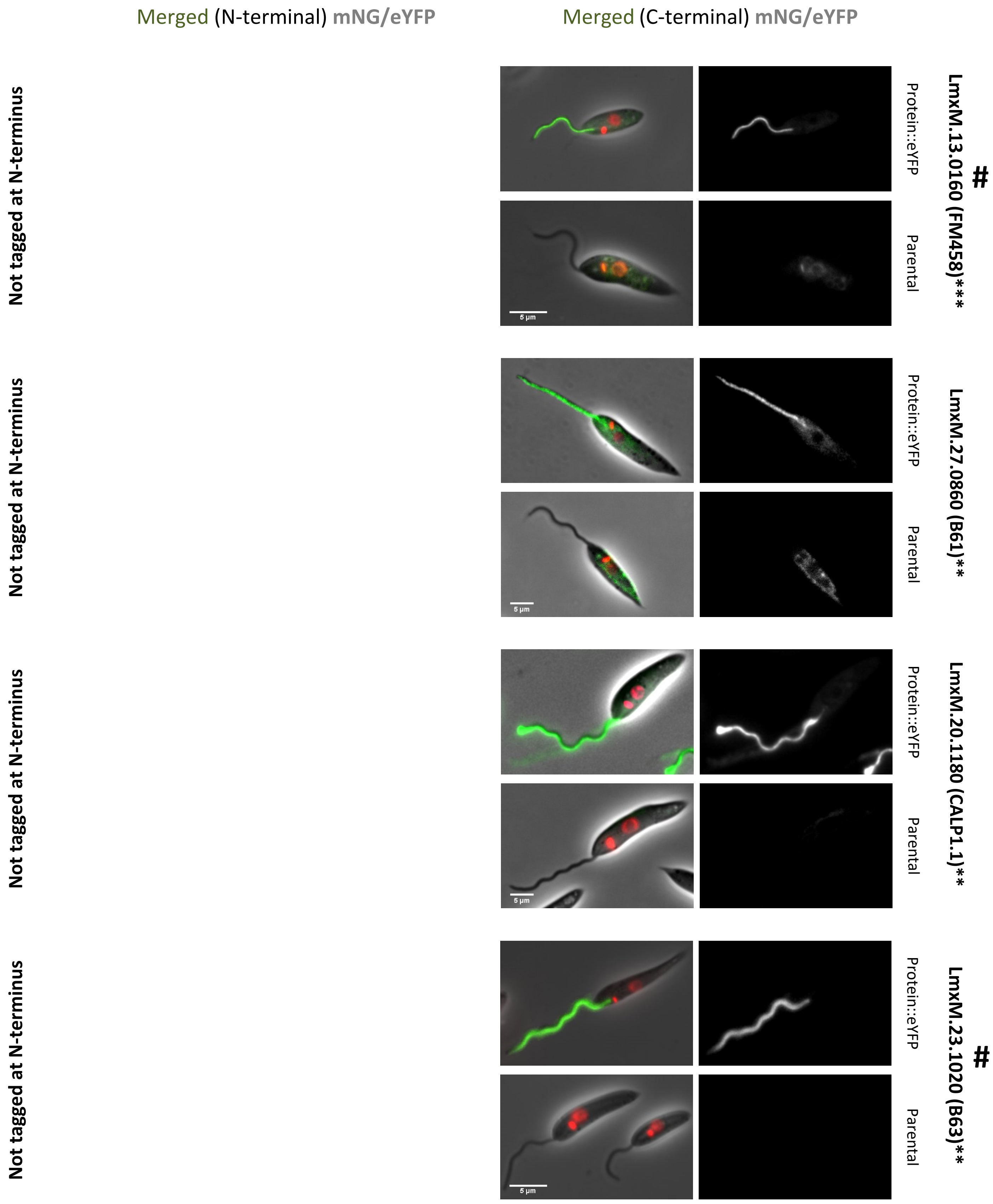

Merged (N-terminal) mNG/eYFP
Merged (C-terminal) mNG/eYFP
Not tagged at N-terminus
Protein::eYFP
Parental
LmxM.13.0160 (FM458)**
#
Not tagged at N-terminus
Protein::eYFP
Parental
LmxM.27.0860 (B61)**
Not tagged at N-terminus
Protein::eYFP
Parental
LmxM.20.1180 (CALP1.1)**
Not tagged at N-terminus
Protein::eYFP
Parental
LmxM.23.1020 (B63)**
#
5 µm

Merged (N-terminal) mNG/eYFP

Merged (C-terminal) mNG/eYFP

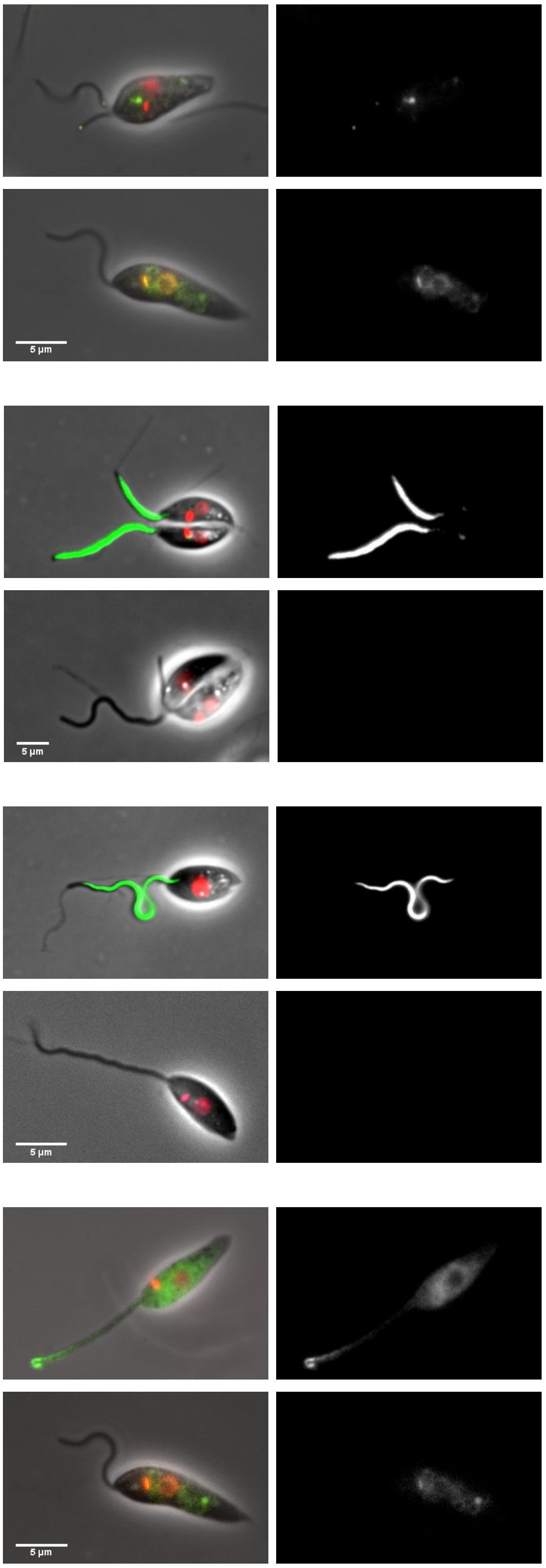

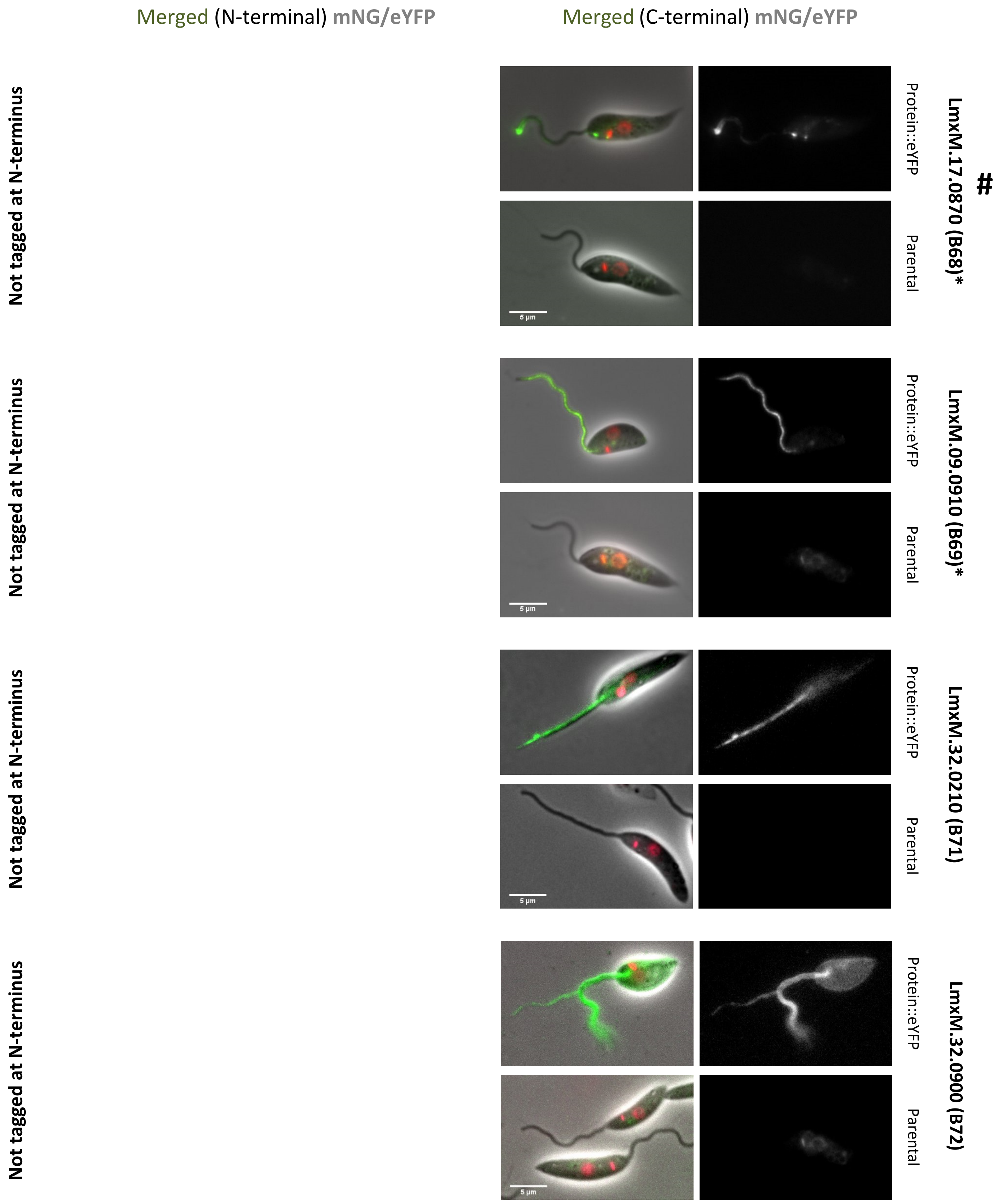

Merged (N-terminal) mNG/eYFP
Merged (C-terminal) mNG/eYFP
Not tagged at N-terminus
LmxM.17.0870 (B68)*
#
Protein::eYFP
Parental
5 µm
Not tagged at N-terminus
LmxM.09.0910 (B69)*
Protein::eYFP
Parental
5 µm
Not tagged at N-terminus
LmxM.32.0210 (B71)
Protein::eYFP
Parental
5 µm
Not tagged at N-terminus
LmxM.32.0900 (B72)
Protein::eYFP
Parental
5 µm

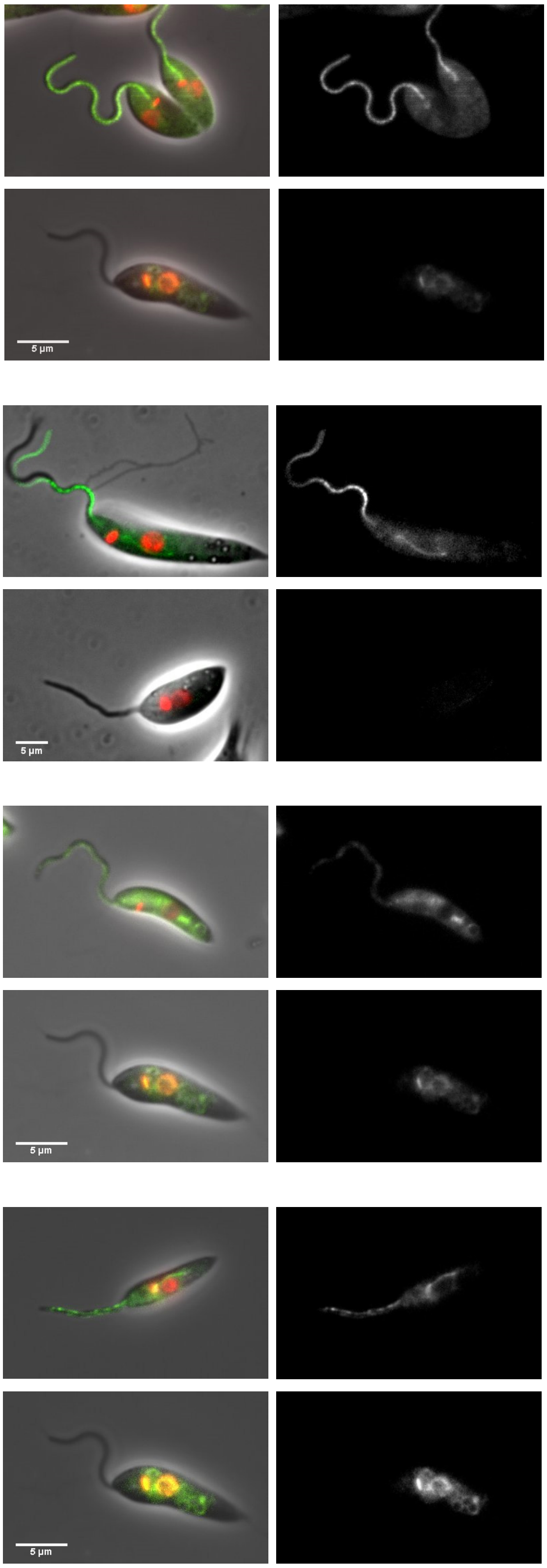

Merged (N-terminal) mNG/eYFP
Merged (C-terminal) mNG/eYFP
Not tagged at N-terminus
LmxM.08_29.1100 (B73)*
Protein::eYFP
Parental
5 µm
Not tagged at N-terminus
LmxM.23_177418 (B75)*
Protein::eYFP
Parental
5 µm
Not tagged at N-terminus
LmxM.29.0930 (B76)
Protein::eYFP
Parental
5 µm
Not tagged at N-terminus
LmxM.36.3180 (B77)
Protein::eYFP
Parental
5 µm

Merged (N-terminal) mNG/eYFP

Merged (C-terminal) mNG/eYFP

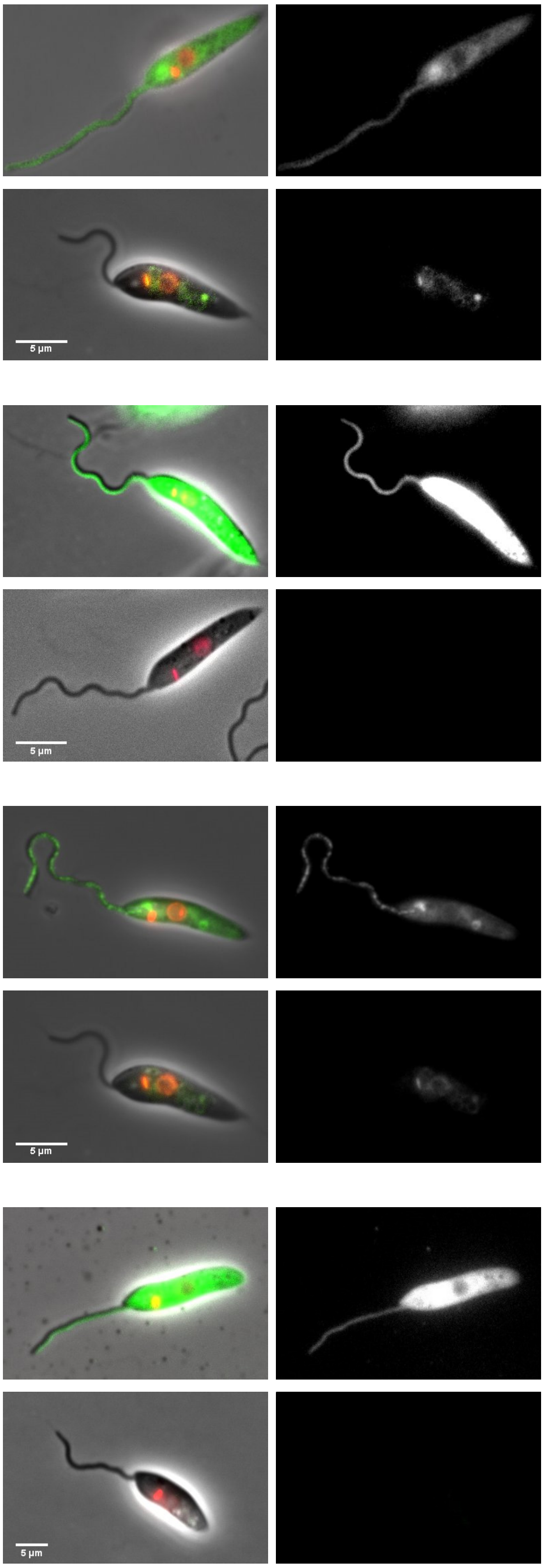

Not tagged at N-terminus

Protein::eYFP

Parental

LmxM.08_29.2440 (B78)*

Not tagged at N-terminus

Protein::eYFP

Parental

LmxM.29.0880 (B79)*

Not tagged at N-terminus

Protein::eYFP

Parental

LmxM.36.2840 (FLAM2)**

Not tagged at N-terminus

Protein::eYFP

Parental

LmxM.08_29.2800 (B81)

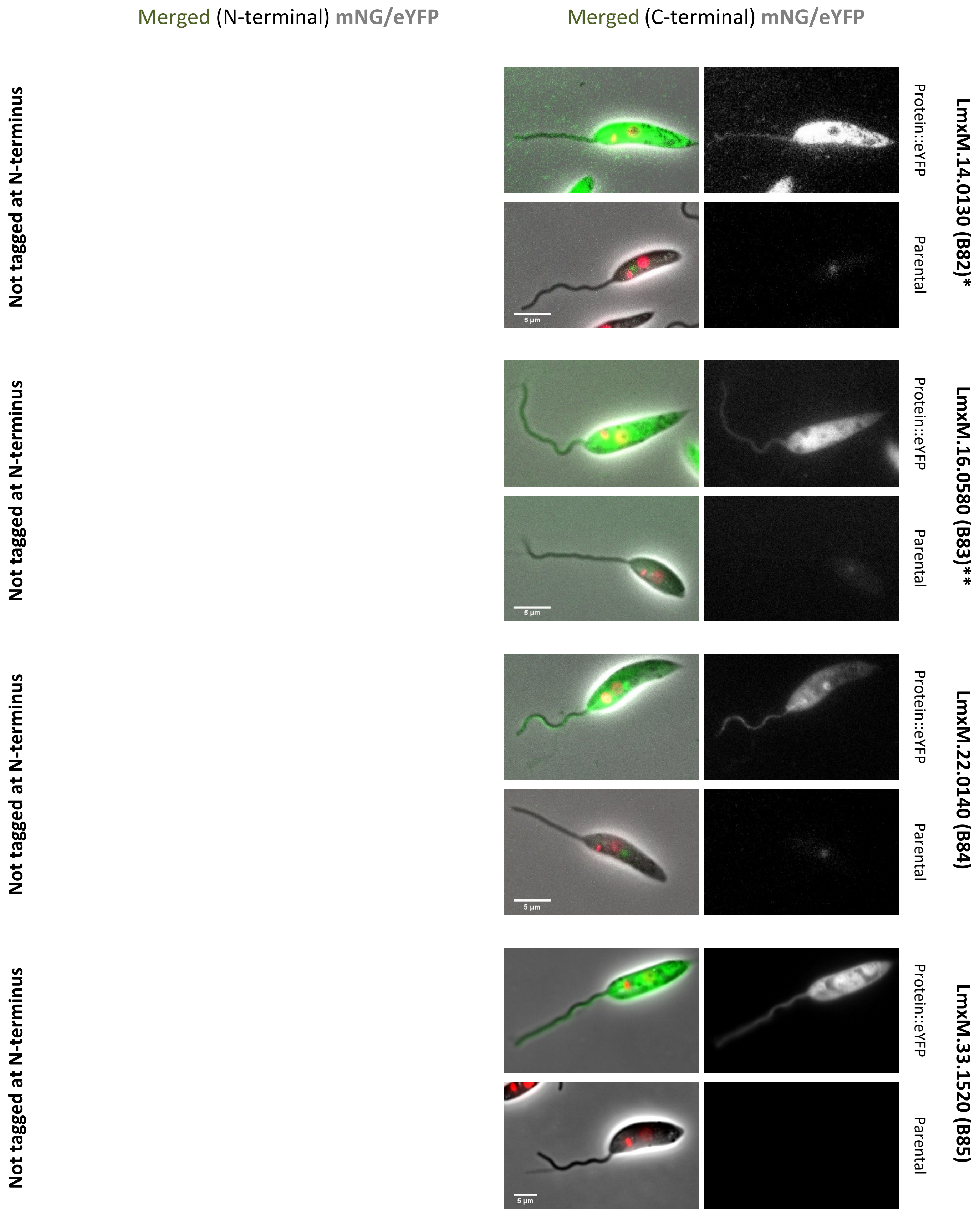

Merged (N-terminal) mNG/eYFP
Merged (C-terminal) mNG/eYFP
Not tagged at N-terminus
Not tagged at N-terminus
Not tagged at N-terminus
Not tagged at N-terminus
Protein::eYFP
Parental
Protein::eYFP
Parental
Protein::eYFP
Parental
Protein::eYFP
Parental
LmxM.14.0130 (B82)*
LmxM.16.0580 (B83)**
LmxM.22.0140 (B84)
LmxM.33.1520 (B85)
5 µm
5 µm
5 µm
5 µm

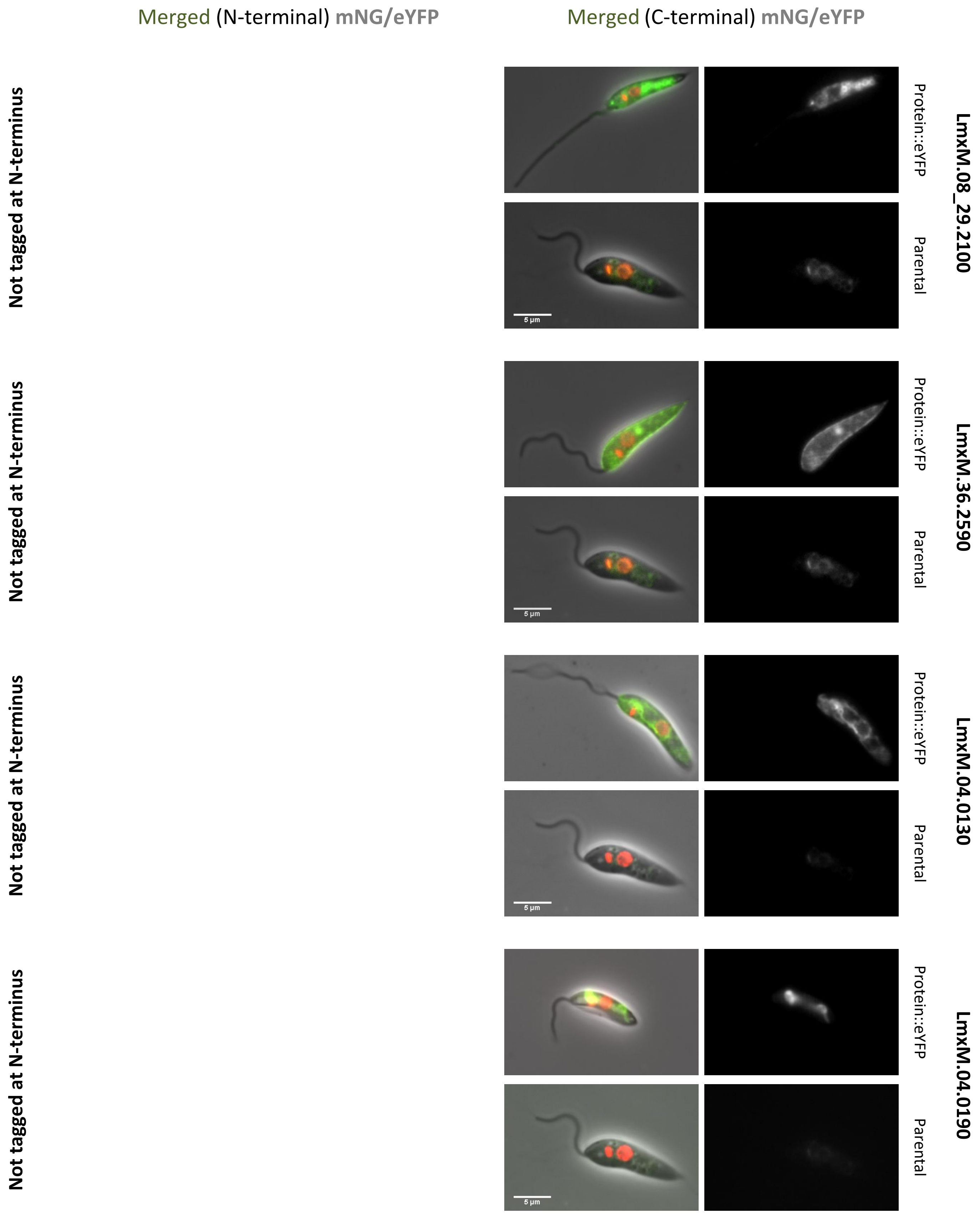
Merged (N-terminal) mNG/eYFP
Merged (C-terminal) mNG/eYFP
Not tagged at N-terminus
Protein::eYFP
Parental
LmxM.08_29.2100
5 µm
Not tagged at N-terminus
Protein::eYFP
Parental
LmxM.36.2590
5 µm
Not tagged at N-terminus
Protein::eYFP
Parental
LmxM.04.0130
5 µm
Not tagged at N-terminus
Protein::eYFP
Parental
LmxM.04.0190
5 µm

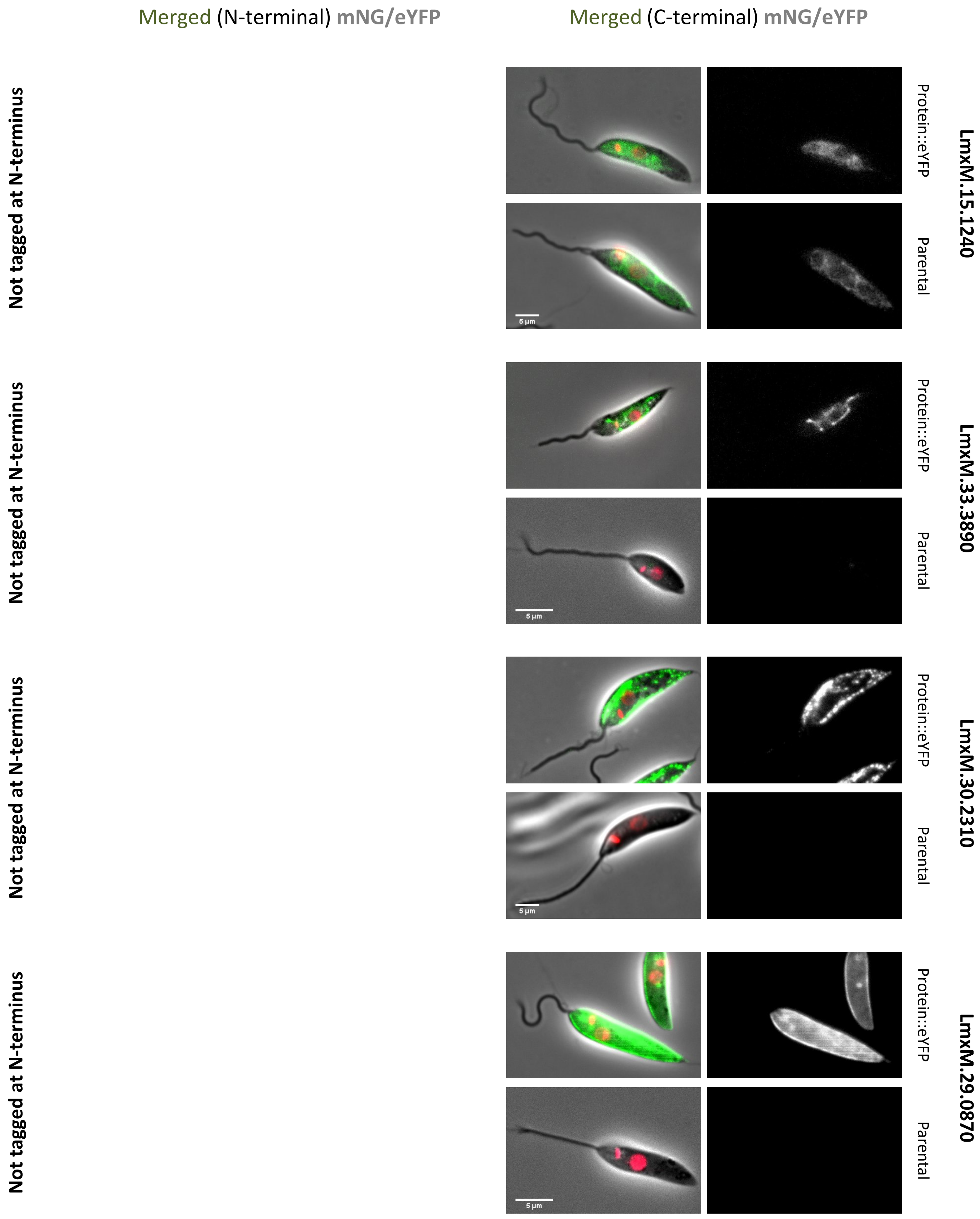
Merged (N-terminal) mNG/eYFP
Merged (C-terminal) mNG/eYFP
Not tagged at N-terminus
Not tagged at N-terminus
Not tagged at N-terminus
Not tagged at N-terminus
Protein::eYFP
Parental
LmxM.15.1240
LmxM.33.3890
LmxM.30.2310
LmxM.29.0870
5 µm

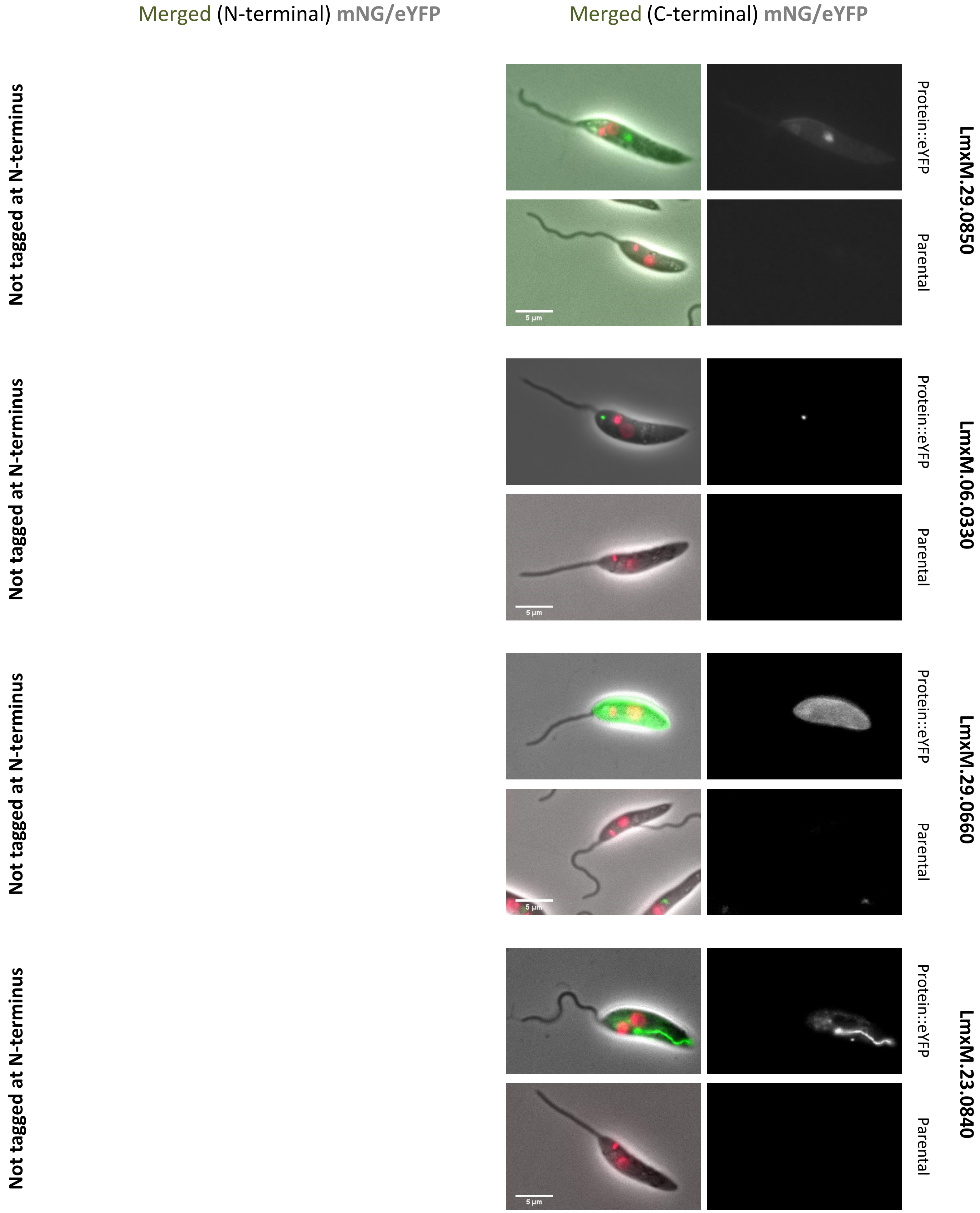
Merged (N-terminal) mNG/eYFP
Merged (C-terminal) mNG/eYFP
Not tagged at N-terminus
Protein::eYFP
Parental
LmxM.29.0850
5 µm
Not tagged at N-terminus
Protein::eYFP
Parental
LmxM.06.0330
5 µm
Not tagged at N-terminus
Protein::eYFP
Parental
LmxM.29.0660
5 µm
Not tagged at N-terminus
Protein::eYFP
Parental
LmxM.23.0840
5 µm

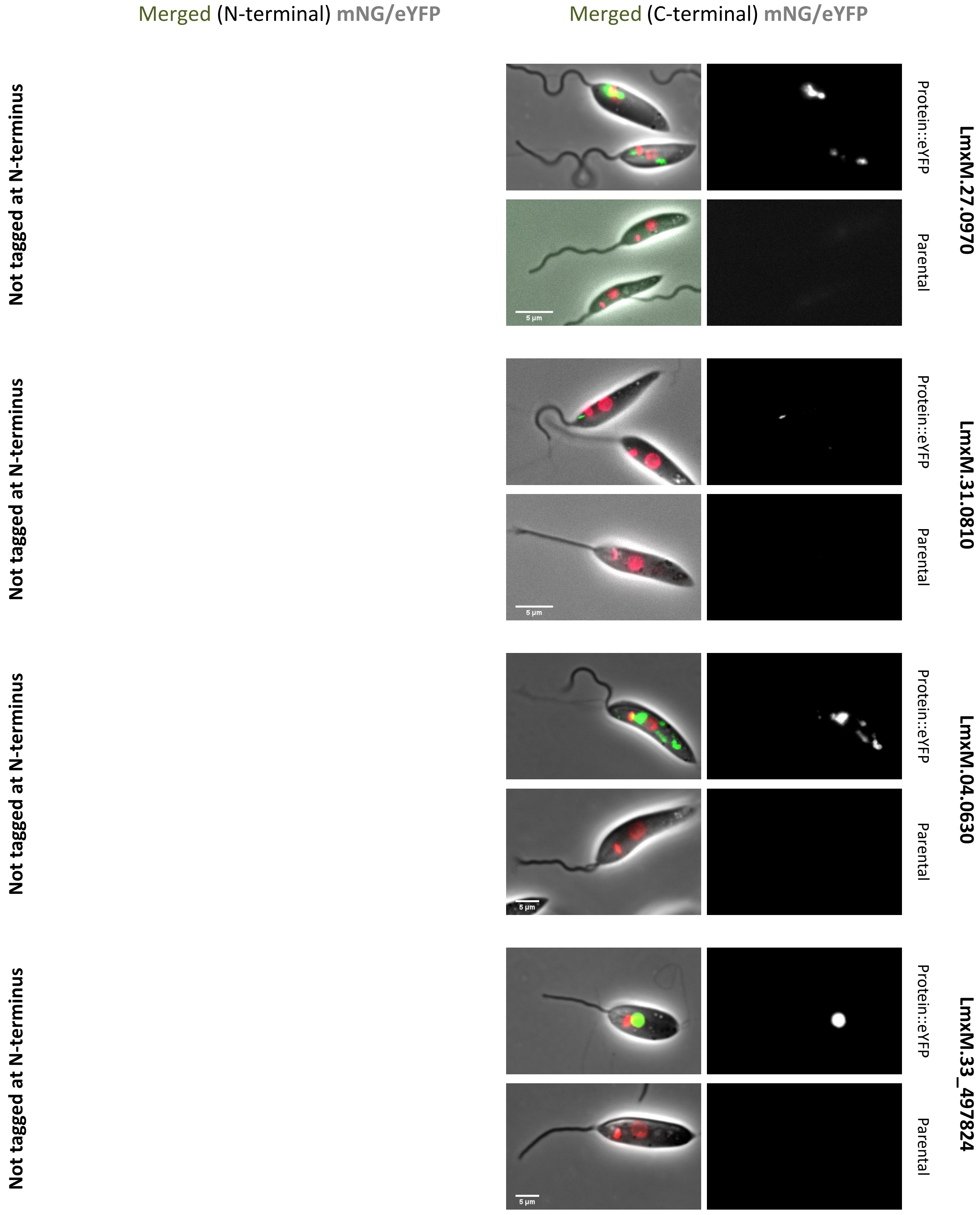
Merged (N-terminal) mNG/eYFP
Merged (C-terminal) mNG/eYFP
Not tagged at N-terminus
Protein::eYFP
Parental
LmxM.27.0970
5 µm
Not tagged at N-terminus
Protein::eYFP
Parental
LmxM.31.0810
5 µm
Not tagged at N-terminus
Protein::eYFP
Parental
LmxM.04.0630
5 µm
Not tagged at N-terminus
Protein::eYFP
Parental
LmxM.33_497824
5 µm

S7 Figure
